# A Highly Cost‐Efficient Large‐Scale Uniform Laminar Plasma Jet Array Enhanced by *V*–*I* Characteristic Modulation in a Non‐Self‐Sustained Atmospheric Discharge

**DOI:** 10.1002/advs.201902616

**Published:** 2020-01-09

**Authors:** Jing Li, Jing Wang, Bingying Lei, Tongyi Zhang, Jie Tang, Yishan Wang, Wei Zhao, Yixiang Duan

**Affiliations:** ^1^ State Key Laboratory of Transient Optics and Photonics Xi'an Institute of Optics and Precision Mechanics of CAS Xi'an 710119 China; ^2^ University of Chinese Academy of Sciences Beijing 100049 China; ^3^ Faculty of Mathematics and Physics Huaiyin Institute of Technology Huaian 223003 China; ^4^ Key Laboratory of Synthetic and Natural Functional Molecule Chemistry of Ministry of Education College of Chemistry and Materials Science Northwest University Xi'an 710127 China

**Keywords:** cost‐efficient plasma jet arrays, discharge stability, material processing, non‐self‐sustained DC discharge, *V*–*I* characteristic modulation

## Abstract

Developing cost‐efficient large‐scale uniform plasma jets represents a significant challenge for high performance in material processing and plasma medicine. Here, a *V*–*I* characteristic modulation approach is proposed to reduce the discharge power and increase the plasma scale and chemical activity in non‐self‐sustained atmospheric direct‐current discharges. The electric field in discharge space is optimized to fundamentally empower simultaneously initiating all discharge cells far below Townsend breakdown potential and stably sustaining each plasma jet at low voltage. These strategies create a crucial step to fabricating a flexible and compact low‐power large‐scale uniform laminar plasma jet array (LPJA) with high activity in cheap argon. The mechanisms behind the discharge enhancement are revealed by combining *V*–*I* characteristic examination and a modulation model. Compared with conventional arrays, this LPJA possesses the widest size (90 mm) and raises its uniformity from 30% to 97%. Comparing different discharge modes shows that the LPJA scale is surprisingly increased nearly by 4 times with the discharge power reduced from 7.4 to 4.8 W. The methodology provides a highly cost‐efficient roadmap to break through the bottleneck of restricting low‐power discharge, large‐gap discharge, large‐scale discharge, parallel‐multi‐electrode discharge, and uniform discharge together. This advance will meet the urgent need for various plasma applications.

## Graphical abstract

A *V*–*I* characteristic modulation approach is proposed in non‐self‐sustained direct‐current discharge to greatly reduce the discharge power and largely increase the plasma scale and chemical activity. In conjunction with the electric field optimization to realize the discharge simultaneity and stability, a highly cost‐efficient large‐scale uniform laminar plasma jet array is fabricated for material processing and plasma medicine.

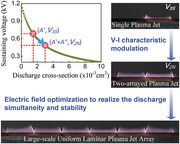

## 1 Introduction

Nonequilibrium atmospheric‐pressure plasmas have been extensively used in various practical applications such as material processing, thin film deposition, nanoscience, plasma medicine, biological sterilization, and chemical analysis, due to their prevailing advantage of avoiding the expensive vacuum system.^1^, ^2^, ^3^, ^4^, ^5^, ^6^, ^7^, ^8^, ^9^, ^10^ These plasmas are usually generated in dielectric‐barrier discharge (DBD), direct‐current (DC) glow discharge, microwave discharge, and radio‐frequency discharge.^11^, ^12^, ^13^, ^14^, ^15^, ^16^, ^17^, ^18^, ^19^ Large and uniform plasmas are in urgent need for treating samples because of their high working efficiency. Array design is a promising way to realize this kind of plasmas. Multi‐microhollow cathode configuration, multi‐microcavity framework, needle‐array electrode equipment, and hexagon electrode mesh have been successfully fabricated to form a large‐scale plasma device.^20^, ^21^, ^22^, ^23^, ^24^, ^25^, ^26^ A common feature of these plasma arrays is that the plasmas are almost sustained between the electrodes and the spatial plasma confinement makes it difficult to use these plasma arrays directly for the samples with irregular shape. The plasma jets are quite favorable because they are generated in the open air rather than in a confined space. The plasma jets have been neatly arrayed up in a linear comb shape.^27^, ^28^, ^29^, ^30^, ^31^ And a honeycomb configuration is another effective strategy to generate the plasma jet array.^32^, ^33^, ^34^, ^35^, ^36^ However, an individual plasma jet typically covers only a few mm^2^ and the spacing between each jet segment extends up to several or tens of millimeters and much larger than the plasma jet itself. These structure designs cannot ensure the uniform distribution of plasmas in one or 2D space and partial surface of the treated samples is likely to be left out in plasma processing. Additionally, a high discharge power is usually required to sustain the discharge in the plasma jet array due to its numerous discharge cells. These deficiencies extremely hinder further improvement of the working efficiency and quality of plasma device. As another important factor dominating the working efficiency, plasmas chemical activity can be enhanced by jet‐to‐jet coupling, preionization, floating electrode, and external magnetic field.^34^, ^37^, ^38^, ^39^, ^40^ But these schemes are only suitable for improving a single plasma jet. Up to date, almost no methods have been proposed to improve the performance of a whole plasma jet array due to its discharge complication. Moreover, when pursuing the commercial value, the cheap argon is desirable. But the constriction of discharge channel into filament at atmospheric pressure prevents generating large‐scale plasmas.^41^, ^42^, ^43^ Thus, developing low‐cost and low‐power large‐scale uniform plasma jet array with high activity is considerably challenging. How to break through low‐power discharge, large‐gap discharge, large‐scale discharge, parallel‐multi‐electrode discharge, and uniform discharge together proves to be the bottleneck of restricting generation of large and uniform plasmas.^32^, ^33^, ^34^, ^35^, ^36^

To overcome the bottleneck problem, this paper demonstrates a new *V*–*I* characteristic modulation enhanced plasma approach in a non‐self‐sustained discharge, based on which a high‐performance laminar plasma jet array (LPJA) with its width up to 90 mm is developed. It is the first time that we use this unique modulation method to reduce the discharge power and increase both the plasma jet length and chemical activity in an array. The electric field in discharge space is optimized to fundamentally solve the problem of failing to simultaneously initiate all the DC discharge cells far below the Townsend breakdown potential and stably sustain each of the plasma jets at a low voltage. The discharge simultaneity and stability create a crucial step to produce a plasma jet array with large scale. We also give special attention to the plasma uniformity, as well as the compactness and flexibility of the device, through alternating anode–cathode arrangement and independent switch settings. Our work shows a great potential to meet the urgent need for various plasma applications.

## 2 Proposing the Model of *V*–*I* Characteristic Modulation

**Figure 1:**
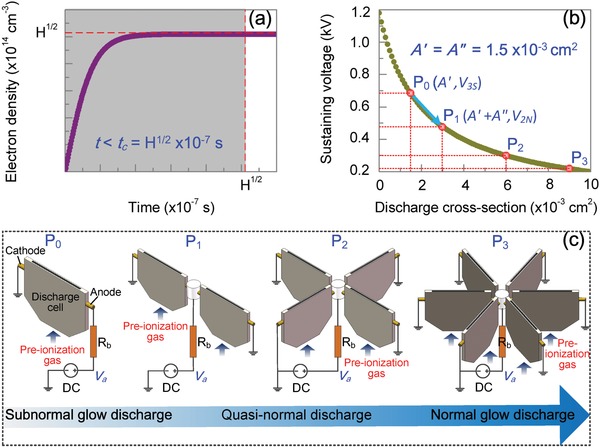
Illustration of the *V*–*I* characteristic modulation model. a) Temporal evolution of electron density in a non‐self‐sustained discharge. b) Variation of the sustaining voltage with the discharge cross‐section. c) Schematic diagram of the *V*–*I* characteristic modulation strategy. The case at the operating point P_0_ means a subnormal glow discharge occurring in a single discharge cell with a higher sustaining voltage and a smaller discharge current. With the output voltage of the DC power supply unvaried, arranging two, four, and six discharge cells in parallel, which respectively corresponds to the cases at the operating points P_1_, P_2_, and P_3_, is an effective way to increase the discharge cross‐section and realize the discharge mode transition.

To ignite a non‐self‐sustained discharge and sustain it stably, an external ionizer is required to provide the preionization that balances the electron losses. The electron density is expressed as a function of time after turning on the external ionizer^44^

(1) $$n_{\text{e}}(t)\;\;=\;\;{\left(\frac{\varphi }{\beta }\right)}^{1/2}\cdot \;\;\frac{\exp\left[{\left(\varphi \beta \right)}^{1/2}t\right]-\exp\left[-{\left(\varphi \beta \right)}^{1/2}t\right]}{\exp\left[{\left(\varphi \beta \right)}^{1/2}t\right]+\exp\left[-{\left(\varphi \beta \right)}^{1/2}t\right]}$$

where φ is the preionization rate of external ionizer and β is the recombination coefficient. Recently obtained experimental results show that the electron density is usually on the order of 10^14^ cm^−3^ in atmospheric‐pressure argon DC discharges sustained by an external ionizer.^45^, ^46^, ^47^ Under the condition that β takes a typical value of 1 × 10^−7^ cm^3^ s^−1^,^43^ φ is estimated to be on the order of 10^21^ cm^−3^s^−1^. Thus, φ can be expressed as $H\;\;\times \;\;{\text{10}}^{\text{21}}\;{\text{cm}}^{-\text{3}}\;{\text{s}}^{-\text{1}}$, where 0 < *H* < 10. Based on Equation (1), the temporal evolution of electron density is given in **Figure** **1**a. It is found that *n*_e_(*t*) grows with time and approaches a steady value $n_{\text{s}}\;\;={\left(\varphi \text{/}\beta \right)}^{1/2}=\;\;H^{\text{1/2}}\times \;\;10^{14}\;{\text{cm}}^{-\text{3}}$, when the time tends to be the characteristic time $t_{\text{c}}=\;\;{\left(\varphi \beta \right)}^{-1/2}=\;\;H^{\text{1/2}}\times \;\;{\text{10}}^{-\text{7}}\;\text{s}$.

It is generally accepted that with increasing the output voltage of the DC power supply, the DC discharge will be transformed from a Townsend discharge to a normal glow one via a subnormal region.^48^ In the subnormal glow mode, the size of cathode spot is on the order of several micrometers and a thin discharge channel bridges the two electrodes.^43^ Since the loss of charged particles in the lateral direction is harmful for multiplication due to free diffusion of electrons to the side walls, the average electron density is relatively low. To increase the average electron density for high plasma chemical activity, the discharge is usually allowed to work in a normal/abnormal mode by further raising the output voltage. But this manipulation brings about another harmful factor, i.e., generation of considerable Joule heat both in the discharge cell and in the ballast resistor because of the increase in the discharge current. The most desirable way is realizing a normal/abnormal glow discharge at a smaller discharge current. In DC discharge, the sustaining voltage *V* follows the equation^44^

(2) $$V\;\;=\;\;\frac{V_{\text{a}}d}{d\;\;+\;\;A\;\;\cdot \bar{n_{\text{e}}}e\mu_{\text{e}}\cdot R_{\text{b}}}$$

where *e* is the electron charge, μ_e_ is the electron mobility, and *d* is the electrode spacing. It follows from Equation (1) that as for the non‐self‐sustained DC discharge, the electron density, as well as its average value $\bar{n_{\text{e}}}$, is largely determined by φ and changed little in the subnormal transition region.^49^ Thus, when the output voltage *V*_a_ and ballast resistor *R*_b_ are given, *V* will decrease with increasing the discharge cross‐section *A*, with the relationship shown in Figure 1b. Here, in order to conveniently compare with the experimental results, we assign 1180 V to *V*_a_, 50 kΩ to *R*_b_, 1.85 × 10^14^ cm^3^ to $\bar{n_{\text{e}}}$, and 1.5 cm to *d* by referring to the experimental electrical parameters at the operating point 3S demonstrated in the later section. It should be noted that the reduction of sustaining voltage with an approximately constant electron density means that loss of charged particles in the lateral direction slows down and the discharge is likely to transform from the subnormal mode to a normal one. The increase of cross‐section can be fulfilled by enlarging the cross‐section of the discharge cell itself. Figure 1c indicates that arranging several discharge cells in parallel is another effective way to increase the discharge cross‐section and realize the discharge mode transition. Arranging two DC discharge cells in parallel is the most simple case, where the total discharge current and sustaining voltage are expressed as follows^44^

(3) $$\{\begin{array}{c} I\;\;=\;\;\left(V_{\text{a}}-\;\;V\right)\text{/}R_{\text{b}}=\;\;\left(A^{\prime }\;\;+\;\;A^{\prime \prime }\right)\cdot \;\;\bar{n_{\text{e}}}e\mu_{\text{e}}E\\ V\;\;=\;\;\frac{V_{\text{a}}d}{d\;\;+\;\;\left(A^{\prime }\;\;+\;\;A^{\prime \prime }\right)\cdot \;\;\bar{n_{\text{e}}}e\mu_{\text{e}}\cdot R_{\text{b}}}\;\;\;\;\;\;\;\;\;\;\;\;\;\;\;\;\;\;\;\;\;\;\;\;\;\;\;\;\;\;\;\;\;\;\;\;\;\;\;\;\;\;\;\;\;\;\; \end{array}$$

Here, *A*′ and *A*′′, respectively, represent the discharge cross‐sections of the two discharge cells and *A*′ = *A*′′ = 1.5 × 10^−3^ cm^2^. We assume that the discharge at a higher sustaining voltage *V*_3S_ is a subnormal one, which occurs at a smaller discharge current *I*_3S_. When the discharge cross‐section increases from *A*′ to *A*′ + *A*′′, the sustaining voltage will be abruptly reduced from *V*_3S_ = 680V to *V*_2N_ = 478V. The operating point shifts from P_0_ to P_1_. Referring to the equation $I\;\;=\;\;(V_{\text{a}}-\;\;V)\text{/}R_{\text{b}}$, we find that the total discharge current *I* increases in the parallel circuit. But, the discharge current flowing through each discharge cell decreases to some extent, which is ascribed to the decreasing electric field *E* between the electrodes as a result of the decreasing sustaining voltage.^44^ Thus, we can obtain the following relationship

(4) $$\{\begin{array}{c} I_{3\text{S}}<I_{2\text{N}}<2I_{3\text{S}}\;\;\;\;\;\;\;\;\;\;\;\;\;\;\;\;\;\;\;\;\;\;\;\;\;\;\;\;\;\;\;\;\;\;\;\\ I_{3\text{S}}=\;\;A^{\prime }\cdot \;\;\bar{n_{\text{e}3\text{S}}}e\mu_{\text{e}}E_{3\text{S}}\;\;\;\;\;\;\;\;\;\;\;\;\;\;\;\;\;\;\;\;\;\;\\ I_{2\text{N}}=\left(A^{\prime }\;\;+\;\;A^{\prime \prime }\right)\cdot \bar{n_{\text{e2N}}}e\mu_{\text{e}}E_{\text{2N}} \end{array}$$

where *I*_2N_ is the sum of discharge current of the two discharge cells in the parallel circuit. For each of the discharge cells, the discharge current equals $I_{2\text{N}}\text{/}2$. To sustain a discharge with the initial value *I*_3S_, it is necessary to increase the discharge current by raising the output voltage. Increasing the output voltage further raises the possibility of the discharge transforming into a normal/abnormal mode.

It is concluded that the *V*–*I* characteristic modulation model proposed above includes two key steps, i.e., 1) arranging non‐self‐sustained DC discharge cells in parallel with the output voltage unvaried, and 2) raising the output voltage until the discharge current increases to the initial value. This model succeeds in realizing a normal/abnormal glow discharge with a higher average electron density at a smaller discharge current, which creates a new theoretical roadmap for the enhanced gas discharge basic knowledge.

## 3 Results and Discussion

### 3.1 Improving the Discharge Stability and Plasma Uniformity

#### 3.1.1 Improving the Discharge Stability by Laminar Flow Control

**Figure 2:**
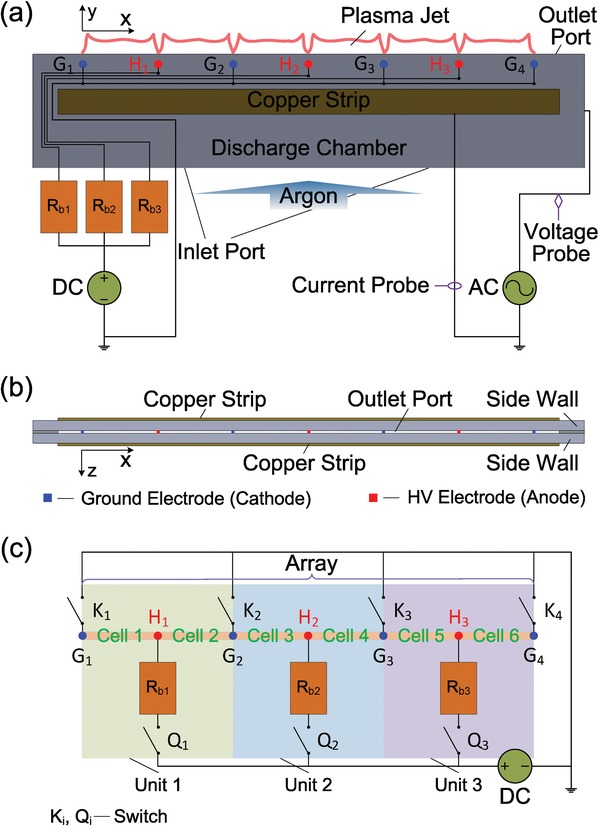
Design strategy of the LPJA. a) The front view of the device. b) The vertical view of the device. c) Equivalent circuit of DC discharge in the device.

Based on the *V*–*I* characteristic modulation model, a LPJA is developed in a non‐self‐sustained DC discharge. This device, as shown in **Figure** **2**a, comprises two main parts: 1) a main discharge part linearly integrated by three discharge units or six DC discharge cells in a nested parallel circuit and 2) a DBD external ionizer used to provide preionization. The detailed experimental setup is described in the Experimental Section. As for the LPJA, the flow characteristic of feeding gas at the outlet of discharge chamber can be evaluated by the Reynolds number^50^

(5) $$R_{\text{e}}=\;\;D_{\text{e}}\cdot v_{\text{g}}\text{/}\nu$$

Here, ν is the argon kinematic viscosity and *D*_e_ is the equivalent diameter of the outlet with an inner rectangular cross‐section, which can be expressed as 4D⋅L/2(D  +  L). At the argon flow of 13 L min^−1^, the Reynolds number *R*_e_ is estimated to be 306 and much less than the critical Reynolds number (*R*_eC_ = 2300) for the transition from the laminar to turbulent regime.^50^ This means the plasma jet array keeps laminar flow, which is helpful for stabilizing the plasma. When a quartz tube with the same inner cross‐sectional area is concerned, the inner diameter *D*_in_ is 8 mm and the Reynolds number *R*_e_′ is estimated to be 2449. This value exceeds the critical Reynolds number and a turbulent gas flow is formed in the quartz tube, which is harmful for stabilizing the plasma. Thus, the flat duck‐mouth shaped outlet, designed here, contributes much to the plasma stability at a higher gas flow rate.

#### 3.1.2 Improving the Discharge Stability by Electric Field Optimization

Here, the DBD, as a preionization source, contributes much to simultaneously initiating the DC discharge cells, but it does not guarantee the discharge stability. The discharge stability, as well as the discharge simultaneity, is fundamentally determined by the electric fields across the gas gaps. The electric field difference between discharge cells unavoidably exists due to the manufacturing errors, which severely restricts the discharge simultaneity and stability. Decreasing or eliminating this difference is an effective route to improve the discharge. Generally, planar electrodes are usually used in the discharge and a stronger electric field is formed near the electrodes due to the effects of edge and corner. The edge and corner effects will also give rise to a larger electric field difference between discharge cells. The discharge instability due to the electric field difference prevents generating a plasma jet array with a larger scale,^46^ especially when all the discharge cells are powered by a single electric source. To overcome this deficiency, we improve the electrode geometry and electrode configuration in this work, where the planar electrodes are replaced by cylindrical ones with their cylindrical surfaces facing to each other. Avoiding the edge and corner effects as far as possible, the cylindrical electrodes not only weaken the electric field near the electrodes but also reduce the electric field difference between discharge cells with different gas gaps.

**Figure 3:**
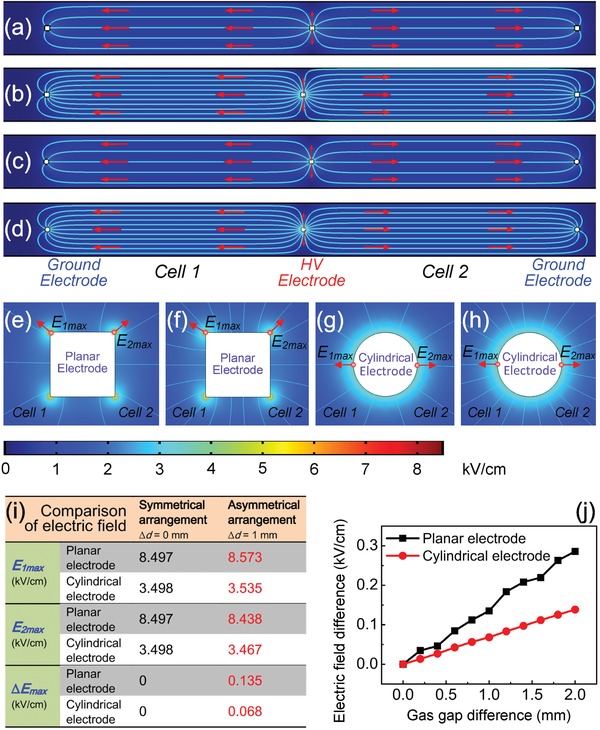
Simulation of the electric field in a discharge unit by solving the Poisson equation. The spatial distributions of electric field in the two adjacent discharge cells equipped with three planar electrodes in a) symmetrical arrangement (Δ*d* = 0) and b) asymmetrical arrangement (Δ*d* = 1 mm). The spatial distributions of electric field in the two adjacent discharge cells equipped with three cylindrical electrodes in c) symmetrical arrangement (Δ*d* = 0) and d) asymmetrical arrangement (Δ*d* = 1 mm). e–h) Shows the enlarged images of field distribution around the central HV electrode in (a–d), respectively. i) Comparison of the electric field of two adjacent discharge cells in the two cases of the planar and cylindrical electrodes. j) Plots of the electric field difference between the two adjacent discharge cells as a function of the gas gap difference in the two cases of the planar and cylindrical electrodes.

To clarify the electric field optimization more clearly, the spatial distributions of electric field in the discharge unit equipped with planar electrodes and cylindrical electrodes were comparatively analyzed by solving the Poisson equation in the *x*–*y* plane, with the results shown in **Figure** **3**. For simplicity, the cross‐sections of the plane electrodes and the cylindrical electrodes are considered to be a square (0.3 mm side length) and a circle (0.3 mm diameter), respectively. The voltage applied on the high voltage (HV) electrode is 900 V (the breakdown potential in the experiment). The local maximum of electric field is used to demonstrate the change of electric field. It follows from Figure 3a,c that a symmetrical profile with respect to the central HV electrode is observed and a same electric field distribution is presented in the discharge cell 1 and cell 2, where the discharge cells have an equal gas gap (*d*_1_ = *d*_2_ = 15 mm) due to the symmetrical electrode arrangement for both the plane electrodes and the cylindrical electrodes. The enlarged images of field distribution around the central HV electrode for the plane electrodes and cylindrical electrodes are shown in Figure 3e,g, respectively. The local maximums of electric field (*E*_1max _, *E*_2max _) are located at the start point of red arrows in the discharge cell 1 and cell 2 for the two different electrode geometries. The red arrows point to the directions of electric field. It is found from the table (Figure 3i) that *E*_1max _ = *E*_2max _ = 8.497 kV cm^−1^ for the plane electrodes and *E*_1max _ = *E*_2max _ = 3.498 kV cm^−1^ for the cylindrical electrodes. Comparing the electric fields in the two different electrode geometries shows that the electric field drops sharply with the cylindrical electrodes used. When there is a deviation (Δ*d* = |*d*_1_ − *d*_2_| = 1 mm) between the gas gaps because of the asymmetrical electrode arrangement, the electric field exhibits an asymmetric spatial distribution in the discharge unit for both the two cases of plane electrodes (Figure 3b) and circle electrodes (Figure 3d). Here, we assume that the sum of the two gas gaps remains constant, i.e., *d*_1_ + *d*_2_ = 30 mm. The images of field distribution around the central HV electrode are also enlarged and shown in Figure 3f (planar electrodes) and Figure 3h (cylindrical electrodes). Similar to the cases in Figure 3e,g, the red arrows indicate the direction and location of the local maximums of electric field in the two discharge cells. As seen in the table (Figure 3i), for the plane electrodes, *E*_1max _ = 8.573 kV cm^−1^ and *E*_2max _ = 8.438 kV cm^−1^. The electric field difference Δ*E*_max _ = |*E*_2max _ − *E*_1max _| = 0.135 kV cm^−1^. For the cylindrical electrodes, *E*_1max _ = 3.535 kV cm^−1^ and *E*_2max _ = 3.467 kV cm^−1^. The difference between *E*_1max _ and *E*_2max _ is 0.068 kV cm^−1^. The electric field difference is also considerably reduced for the cylindrical electrodes. With increasing the gas gap difference in the range of 0–2 mm, the electric field difference increases approximately linearly in the two cases of the planar and cylindrical electrodes (Figure 3j). Comparing the two cases indicates that cylindrical electrode makes the electric field difference drop to half of the value obtained in the case of planar electrodes. The considerable reduction of electric field difference between the discharge cells plays an important role in simultaneously initiating the two DC discharge cells and subsequently stabilizing the discharge. The *V*–*I* characteristic modulation model indicates that the decrease of sustaining voltage with increasing the discharge cross‐section is accompanied by the discharge transition from subnormal to normal mode. Another remarkable characteristic is that the cylindrical electrodes undoubtedly increase the effective discharge cross‐section, compared to the planar ones. This promotes the discharge transition, which is beneficial to the discharge stability and plasma chemical activity in the non‐self‐sustained discharge.^51^

#### 3.1.3 Improving the Plasma Scale and Uniformity and the Structure Compactness by Alternating Anode–Cathode Arrangement

**Figure 4:**
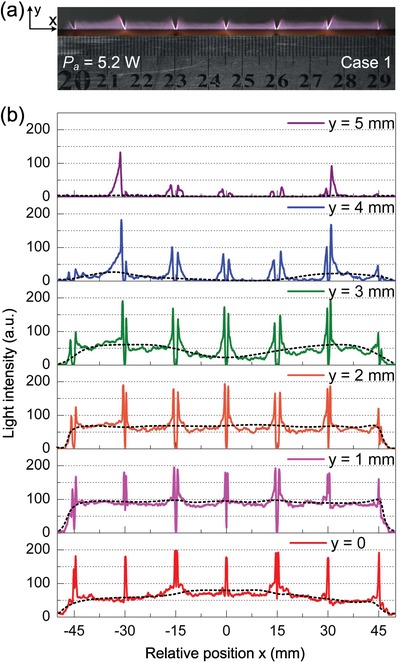
Physical and optical characteristics of the LPJA. a) The image of the LPJA. b) The transverse distribution of light intensity of the LPJA at different lengths with *y* = 0, 1, 2, 3, 4, and 5 mm, respectively.

Just because of the electric field optimization, a simultaneous and stable discharge is realized for the two adjacent DC discharge cells in a discharge unit. The discharge simultaneity and stability create a crucial step for assembling multiple discharge units in parallel to produce a plasma jet array with a larger scale. This assembling is realized by arranging the anodes and cathodes in an alternating manner. As shown in **Figure** **4**a, an array of large stable plasma jets is achieved with its width up to 90 mm. In spite of the large transverse dimension, the jet length reaches up to 5 mm with the discharge power of 31 W and argon flow of 13 L min^−1^.

The light intensity of the LPJA (Figure 4b) was examined along the *x* or transverse direction at different lengths with *y* = 0, 1, 2, 3, 4, and 5 mm, respectively. It is found that the LPJA emits much stronger light at the electrodes than in other regions. Excluding the light at the electrodes, the transverse profile of light intensity takes a transition from convex shape, via flat side, to concave outline with increasing the length. At the length of *y* = 0, a center‐advantage distribution of light intensity is presented just above the outlet of the discharge chamber, because more excited atoms are brought out through the central part of the outlet by a higher gas flow existing there. With the length increased to *y* = 1 mm, as compared with the case at *y* = 0, the light intensity is increased as a whole and distributed uniformly along the transverse direction, not considering the strong light at the electrodes. When the length is further increased to *y* = 2 mm, the light intensity presents an approximately uniform transverse distribution with its magnitude reduced to some extent. For the case at *y* = 3 mm, the light intensity gradually decreases as a whole, with its magnitude higher at edges than in the center. At the length of *y* = 4 mm, excluding the strong light at the electrodes, the light intensity tends to first disappear from the center. When the length rises up to 5 mm, the light intensity almost cannot be detected except that at the electrodes.

In order to characterize this LPJA more quantitatively, we introduce a relative plasma uniformity coefficient, which is defined as the ratio of the sum of transverse linear span (or cross‐sectional area) of each plasma jet to the transverse linear span (or cross‐sectional area) of the whole plasma jet array. As for the conventional plasma jet arrays, in most cases, the electrodes are arranged in the similar or same manner as the DBD, where the plasma jets are isolated by the electrodes and dielectrics placed between each discharge cell. Additionally, due to the rapid quenching of plasma in the open air,^52^, ^53^ the plasma jets fail to effectively expand in the radial direction and overlap with each other to form a large uniform plasma jet array. Thus, the plasma uniformity usually remains at a low level of about 30%.^27^, ^28^, ^29^, ^30^, ^31^, ^32^, ^33^, ^34^, ^35^, ^36^ In our case, the plasma uniformity is raised up to 97.0% and 94.5% at the length of 1 and 2 mm, respectively. This great improvement of plasma uniformity is ascribed to the alternating anode–cathode arrangement. Additionally, the alternating anode–cathode arrangement diminishes the distance between each discharge cell to the utmost degree and makes all the discharge cells closely linked with one another. This distinctive design ensures a largest plasma generated in a limited space and exhibits a compact feature.

### 3.2 Reducing Discharge Power and Increasing Plasma Scale and Plasma Activity

#### 3.2.1 *V*–*I* Characteristic Examination and Flexibility Design

**Figure 5:**
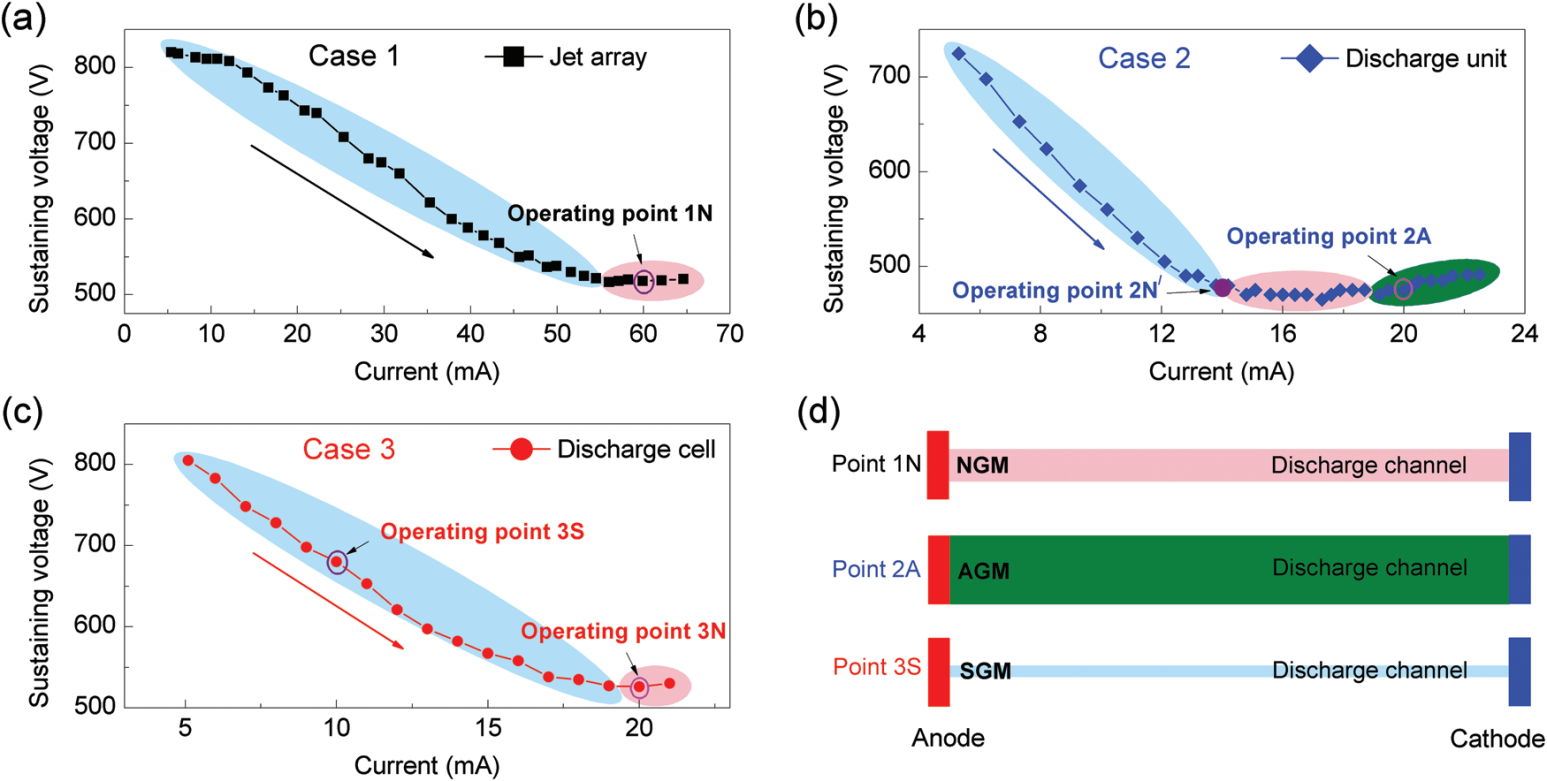
*V*–*I* characteristics of DC discharge for three discharge arrangements. a) A plasma jet array integrated by six DC discharge cells in a nested parallel connection. b) A discharge unit composed of a couple of DC discharge cells in a parallel connection. c) A single DC discharge cell. d) Schematic diagram of the DC discharge structure in three discharge modes, i.e., the normal glow mode (NGM) at the operating point 1N, abnormal glow mode (AGM) at the operating point 2A, and subnormal glow mode (SGM) at the operating point 3S.

The *V*–*I* characteristics of the non‐self‐sustained DC discharge were examined for three different discharge arrangements, i.e., 1) six DC discharge cells arranged in a nested parallel connection, 2) a discharge unit composed of a couple of DC discharge cells in a parallel connection, and 3) a single DC discharge cell, with the results shown in **Figure** **5**a–c, respectively. Here, Case 1 corresponds to the situation that all the independent switches K_i_ and Q_i_ are closed in Figure 2c. Case 2 means that one of the units is selected as the discharge generator. For example, K_2_, K_3_, and Q_2_ are closed and others are opened. As for Case 3, one of the cells is acted as the discharge generator. For example, K_2_ and Q_1_ are closed and others are opened. It follows from Figure 5a that for Case 1, the sustaining voltage first decreases with the current in a large range of 5–55 mA and then approximately remains unchanged with the current further increased from 55 to 65 mA. At the operating point 1N with the sustaining voltage of 519 V and current of 60 mA, we achieved a six‐arrayed plasma jet, as shown in Figure 4a. The *V*–*I* characteristic for Case 2 is shown in Figure 5b. It is found that the sustaining voltage drops with the current in the range of 5–14 mA, approximately remains constant with the current increased from 14 to 19 mA, and rises slightly when the current is further increased from 19 to 23 mA. With respect to Case 3, the reduction of sustaining voltage with the current is observed in a current scope of 5–19 mA in Figure 5c. With further increasing the current of interest, the sustaining voltage changes little. It is generally accepted that the sustaining voltage at low currents corresponds to the gas breakdown potential.^48^ From the *V*–*I* characteristics of DC discharge for all the cases, the breakdown potential is approximately determined to be 900 V and rather low in contrast with that obtained from the Paschen's curve depicted in Figure S1a (Supporting Information).^44^ The sustaining voltage located in the flat area of *V*–*I* curve for all the cases is around 500 V and far below that without preionization in our previous work.^54^

**Figure 6:**
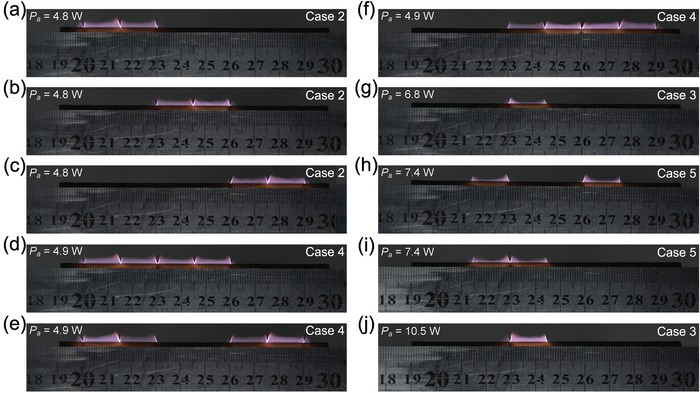
Flexible arrangements for generating desirable plasma jets. a–c) A two‐arrayed plasma jet generated in Case 2, where one of the discharge units works separately. d–f) A four‐arrayed plasma jet generated in Case 4, where any two of the discharge units work together. g,j) A single plasma jet generated in Case 3, where one of the discharge cells works separately. h,i) A two‐arrayed plasma jet generated in Case 5, where any two of the independent discharge cells work together.

In the nested parallel circuit, the LPJA can be flexibly controlled by modulating these independent switches in accordance with the actual demand for local sample treatment and utmost energy saving, as well as for comparing and analyzing the discharge features under different discharge modes but with the same discharge current for each discharge cell. Case 2 (**Figure** **6**a–c) shows a two‐arrayed plasma jet when each discharge unit works independently at the operating point 2A with the sustaining voltage of 475 V and current of 20 mA. Of course, any two of them are also able to work together, with a four‐arrayed plasma jet formed in Case 4 (Figure 6d–f), where the sustaining voltage is 488 V and the current is 40 mA. Additionally, corresponding to Case 3, any of the discharge cells can generate a single plasma jet conveniently (Figure 6g), where the device works at the operating point 3S with the sustaining voltage of 680 V and current of 10 mA. What's more, two independent discharge cells can be selectively combined and Case 5 (Figure 6h,i) shows the examples with the sustaining voltage of 742 V and current of 20 mA.

#### 3.2.2 Reducing Discharge Power and Increasing Plasma Scale by *V*–*I* Characteristic Modulation

For comparison of these plasma jets shown in Figures 4a and 6, the average current for each DC discharge cell is set at 10 mA, except that in Figure 6j. Here, we assume that the current is averaged by the number of discharge cells in operation for all the cases. As for the plasma jet operated at the operating point 3S and shown in Figure 6g, the discharge power is 6.8 W and greater than that for each of the plasma jets shown in Figure 4a. But, it has a shorter length. In addition, the discharge power deposited in each of the plasma jets shown in Figure 6a–c is estimated to be 4.8 W and less than that of the plasma jet in Figure 6g. However, a longer plasma jet is obtained. When two independent and isolated discharge cells work together with the sustaining voltage of 742 V and current of 20 mA, the length of plasma jets presented in Figure 6h maintains the same size as that of plasma jet shown in Figure 6g. It is likely that a longer plasma jet could be obtained provided that two or more adjacent discharge cells work together. As reported previously, a plasma jet could be reinforced through direct jet‐to‐jet coupling by using honeycomb‐structured quartz tube arrays at atmospheric pressure, where the enhancement resulted from the electrical coupling of charged particles in the plasma.^34^, ^55^ To verify this regime in our case, two independent and adjacent discharge cells are arranged to work together, with the two‐arrayed plasma jet obtained in Figure 6i. It is found that the length of the plasma jet does not increase compared to that shown in Figure 6g, although a higher discharge power of 7.4 W is deposited in each of the plasma jets. Accordingly, the enhancement of plasma jets does not originate from the regime of jet‐to‐jet coupling.

Further investigation on the underlying enhancement mechanism and the better interpretation of LPJA would be very useful to improve the performance of the device. Now, let's turn our eyes to the *V*–*I* characteristics of the DC discharge. As shown in Figure 5c, a subnormal glow discharge (or subnormal glow mode, SGM) occurs at the operating point 3S, where the sustaining voltage declines with the current. Since a thin discharge channel is formed between the two electrodes, as illustrated in Figure 5d, only a little part of flowing argon passes through the discharge channel and has a chance to be ionized or excited to a high energy level. This is responsible for generation of a short plasma jet with a high discharge power of 6.8 W (Figure 6g). The discharge at the operating point 1N is operated in a normal glow mode (NGM) (Figure 5a), where the sustaining voltage remains constant with the current. A larger discharge channel (Figure 5d) is developed between the two electrodes and more argon flows through the discharge region and takes part in ionization and excitation. Thus, a longer plasma jet is obtained even with a lower discharge power of 5.2 W. By comparing the plasma jets generated at the operating points 1N and 3S, the influence of discharge mode on plasma performance has been verified in experiments.

As theoretically stated, arranging discharge cells in parallel is an effective way to lower sustaining voltage with the electron density unchanged in a non‐self‐sustained discharge, which allows for the transition of discharge from subnormal to normal mode. At the operating point 3S, the output voltage *V*_a_ = 1180 V and the sustaining voltage *V*_3S_ = 680V. From Figure 1b, it is found that when the output voltage remains unchanged, the sustaining voltage is reduced to *V*_2N_ = 478V after parallel connection of two discharge cells. According to $I=\left(V_{\text{a}}-\;\;V\right)\text{/}R_{\text{b}}$, the total discharge current increases from the initial value *I*_3S_ = 10 mA to *I*_2N_ = 14 mA. But for each discharge cell, the discharge current equals $I_{2\text{N}}\text{/}2\;\;=\;\;7\;\;\text{mA}$. A close observation of Figure 5b indicates that the sustaining voltage 478 V and current 14 mA are just located on the experimentally measured *V*–*I* characteristic curve and labeled as the operating point 2*N*′. From the curve profile, it is found that a normal or quasi‐normal discharge occurs at this operating point, which agrees rather well with the theoretical prediction.

With the output voltage further increased to 1475 V, the discharge current for the discharge unit is increased from 14.0 to 20.0 mA (double of the initial value) and the discharge gets through the normal regime and reaches the abnormal glow mode (AGM) at the operating point 2A, where the sustaining voltage rises with the current. In this discharge mode, the whole cathode has been covered with the discharge and in comparison with the subnormal or normal mode, the discharge occurs in a larger space (Figure 5d). Thus, a larger part of flowing argon passes through the channel and attends ionization and excitation, and a longer plasma jet is produced even with a smaller discharge current of 10 mA and a lower discharge power of 4.8 W for each discharge cell.

In our experiments, with the average current of 10 mA for each of the discharge cells, the sustaining voltage is 680 V for the subnormal discharge at the operating point 3S, 518 V for the normal discharge at the operating point 1N, and 475 V for the abnormal discharge at the operating point 2A. The average electron density $\bar{n_{\text{e}}}$ in the three cases can be estimated from the expression $\bar{n_{\text{e}}}=\;\;\bar{j}\text{/}(E\mu_{\text{e}}e)$,^56^ where $\bar{j}$ is the average current density defined as the current divided by the cross‐section (0.3 mm × 0.5 mm) of the gas gap. $\bar{j}$ equals 6.7 A cm^−2^ for all the cases. The electric field *E* in the discharge is given as the sustaining voltage divided by the gas gap and its value is estimated to be 0.45 kV cm^−1^ for the subnormal discharge, 0.34 kV cm^−1^ for the normal discharge, and 0.32 kV cm^−1^ for the abnormal discharge. Taking the elevated gas temperature of 343 K into account,^44^ the electron mobility μ_e_ is determined to be 4.96 × 10^2^ cm^2^ V^−1^ s^−1^ from the modified equation ${\left(\mu_{\text{e}}p\right)}_{\text{T}}=\;\;T\text{/}T_{0}{\left(\mu_{\text{e}}p\right)}_{{\text{T}}_{\text{0}}}$. With the electron charge *e* = 1.6 × 10^−19^ C, the average electron density $\bar{n_{\text{e}}}$ in the gas gap is 1.85 × 10^14^, 2.43 × 10^14^, and 2.65 × 10^14^ cm^−3^ in turn for the three cases mentioned above. It is seen that the average electron density in the subnormal glow discharge is less than those in other two cases, although the same average current for each of the discharge cells is held in the three different discharge modes. This is the fundamental reason for producing a shorter plasma jet at the operating point 3S. When the DC discharge works in a normal/abnormal mode, a higher average electron density is obtained and a longer plasma jet is achieved. For the normal discharge at the operating point 1N and the abnormal discharge at the operating point 2A, it is assumed that the steady value of electron density ${\left(\varphi \text{/}\beta \right)}^{1/2}$ approximately equals the average electron density and we obtain φ ≈ 6.5 × 10^21^ cm^−3^ s^−1^ and *H* = 2.55. When the 2D variables (φ, *j*) take the values of (6.5 × 10^21^ cm^−3^ s^−1^,6.7 A cm^−2^), the electric field required to sustain the discharge is determined from Equation S8 (Supporting Information) to be 0.33 kV cm^−1^, which is located at the point A on the red curved surface shown in Figure S1c (Supporting Information).^44^

To obtain a plasma jet with a comparable length in an independent discharge cell (Case 3 in Figure 6j), we transform the discharge from subnormal to normal glow mode, with the sustaining voltage decreased to 526 V and current increased to 20 mA at the operating point 3N labeled in Figure 5c. In this case, the discharge power for each cell is 10.5 W and about two times of that at the operating points 1N and 2A. The total consumed power including Joule heat in the ballast resistor is 30.5 W and also increased by two times. It is found that much more energy is consumed in the nonparallel circuit. The overmuch energy consumption sharply increases the requirement for the load capacity of power source.

#### 3.2.3 Increasing Plasma Chemical Activity by *V*–*I* Characteristic Modulation

Case 2 shown in Figure 6b was selected as alternative to make a comparative optical emission spectra (OES) examination in contrast to Case 5 shown in Figure 6i.^44^ Comparing the spectra in the two cases shows that the spectral intensity of the plasma generated in the normal/abnormal glow discharge is approximately increased by two times compared to that in the subnormal glow discharge. This means that the concentration of reactive species in the plasma, as well as the plasma chemical activity, can also be enhanced by modulating the *V*–*I* characteristics of DC discharge. Plasma nonequilibrium characteristics were verified for Case 1 shown in Figure 4a by examining the rotational temperature and vibrational temperature.^44^ Comparing the rotational temperature *T*_rot_ = 640 ± 18 K and the vibrational temperatures *T*_vib_ = 2630 ± 34 K indicates that the LPJA is under nonequilibrium condition, which contributes much to the plasma chemistry enhancement. Spatial examination of the LPJA gas temperature shows that this temperature is far below the rotational temperature, but close to the room temperature, which is beneficial to treating samples susceptible to high temperatures.

Comparative study of the three discharge modes indicates that modulation of *V*–*I* characteristics is an alternative and effective method to improve the plasma performance by increasing the discharge cross‐section and optimizing the discharge mode in a non‐self‐sustained discharge, which exhibits a striking contrast to the conventional approaches, such as jet‐to‐jet coupling, preionization, floating electrode, and external magnetic field. In our previous work,^47^ an external magnetic field *B* was employed to improve the performance of a single plasma jet generated by DC discharge, where the magnetic field was perpendicular to the electric one and the direction of *E* × *B* drift for the magnetized electrons was consistent with the gas flow. In this work, the alternating anode–cathode arrangement makes the electric fields in all the discharge cells not point to the same direction, i.e., one group in *x* direction and the other in −*x* direction. When applying an external magnetic field perpendicular to the electric one, the *E* × *B* drift is upward for one team of discharge cells, and downward for the other,^57^ failing to improve the performance of the plasma jets in the array together. But the *V*–*I* characteristic modulation method overcomes this defect and realizes simultaneous enhancement of all the plasma jets. In conjunction with the electric field optimization, this modulation method successfully breaks through the bottleneck of restricting the generation of low‐power discharge, large‐gap discharge, large‐scale discharge, parallel‐multi‐electrode discharge, and uniform discharge together.

## 4 Conclusion

In summary, a *V*–*I* characteristic modulation approach, as a new enhanced gas discharge theoretical roadmap, is proposed to greatly reduce the discharge power and largely increase both the plasma scale and chemical activity in a non‐self‐sustained atmospheric DC discharge. Increasing the non‐self‐sustained discharge cross‐section gives rise to the transition of discharge from subnormal to normal/abnormal mode, which reduces the loss of charged particles in the lateral direction and leads to the discharge enhancement. The electric field in discharge space is optimized to simultaneously initiate all the DC discharge cells far below the Townsend breakdown potential and stably sustain each of the plasma jets at a low voltage, where the discharge instability is fundamentally eliminated by reducing the electric field difference between discharge cells. Under the support of discharge simultaneity and stability, a nested parallel circuit was designed to produce a LPJA with large scale. In addition, the alternating anode–cathode arrangement ensures a uniform plasma and a compact device. The independent switches provide an important flexibility for the local sample treatment and utmost energy saving. Again by virtue of the nonequilibrium property and low‐temperature feature, this LPJA can be efficiently used in processing various kinds of samples, irrespective of sample size and shape. With these merits, we suggest that our work not only breaks through the bottleneck of restricting the generation of large and uniform plasmas, but also provides a promising and productive approach for developing low‐cost and highly efficient atmospheric plasma sources that are in urgent need for various plasma applications.

## 5 Experimental Section

*Design Strategy of the Laminar Plasma Jet Array*: The LPJA is designed based on the *V*–*I* characteristic modulation model in a non‐self‐sustained DC discharge. This device, as shown in Figure 2a, comprises two main parts: 1) a main discharge part integrated by six DC discharge cells and 2) a preionization part composed of DBD with two parallel copper strips (5 mm × 100 mm) set outside but close to the ceramic discharge chamber (1 mm thickness and 9.8 relative permittivity). As for the main discharge part, four ground electrodes (G_1_, G_2_, G_3_, and G_4_) and three HV electrodes (H_1_, H_2_, and H_3_) are linearly and alternatingly arranged with the same spacing of 15 mm and six DC discharge cells are constructed along the outlet port (the width *D* = 0.5 mm and the length *L* = 100 mm) with 2 mm apart. Here, seven platinum rods with the diameter of 0.3 mm are employed as electrodes with their cylindrical surfaces facing to each other. The electrodes G_1_, G_2_, G_3_, and G_4_ are served as cathodes and connected to the ground via the switches K_1_, K_2_, K_3_, and K_4_, respectively. Acting as anodes, the HV electrodes H_1_, H_2_, and H_3_ are respectively in series with three ballast resistors *R*_b1_, *R*_b2_, and *R*_b3_ (*R*_b1_ = *R*_b2_ = *R*_b3_ = *R*_b_ = 50 *k*Ω) in three independent branches, before they are together connected to a DC power supply with a maximum power of 500 W. These branches are controlled by the switches Q_1_, Q_2_, and Q_3_, respectively. All the switches K_i_ and Q_i_ are independently set to flexibly control the circuit. When all these switches are closed, this circuit design forms three independent discharge units, i.e., Unit 1 consists of Cell 1 and Cell 2 in parallel connection, Unit 2 is composed of Cell 3 and Cell 4 in parallel connection, and Unit 3 consists of Cell 5 and Cell 6 in parallel connection. The three discharge units are again combined into a large discharge array via parallel connection. Thus, the entire circuit shows a nested parallel connection structure. For the preionization part set between the inlet port and the six DC discharge cells, the ceramic sidewalls are used as dielectric of the DBD and the two copper strips are connected to a high‐voltage alternating current transformer (CTP‐2000 K) with the operating frequency *f* ranging from 5 to 20 kHz. In this work, the frequency is fixed at 8.1 kHz. The distance *l* between the DBD and the DC discharge cells is 5 mm.

*Methods for Electrical and Optical Characterization*: High pure argon (99.999%), controlled by a mass flowmeter, enters by the inlet port, flows through the discharge chamber, and gets weakly ionized in the DBD preionization region. The applied voltage between the two copper electrodes and the discharge current were directly measured by using a HV probe (Tektronix P6015A) and a current probe (Tektronix TCP312A), respectively. The waveforms of applied voltage and discharge current were simultaneously recorded by a digital oscilloscope (Tektronix 3054C). Pre‐ionized Ar flows downstream to the main discharge region and there a stable plasma can be generated with a proper DC voltage applied to the three HV electrodes. The plasma is extended as a laminar brush‐shaped jet out of the discharge chamber through the outlet port due to the gas flow. As a result, a large array of laminar plasma jets is presented in front. The plasma generated in the device was imaged with a digital camera (Nikon D5200). The OES from the plasma were measured by using two spectrometers with different resolution. An Avantes spectrometer (AvaSpec‐ULS2048‐USB2) with a moderate resolution of 0.6 nm in the range of 200–1000 nm was used to identify various reactive species in the plasma. Another Avantes spectrometer (AvaSpec‐ULS2048‐3‐USB2) with a high resolution of 0.05 nm in the range of 200–416 nm and 0.3 nm in the range of 415–940 nm was employed to determine the rotational and vibrational temperatures of the plasma jet. Both their fiber probes were placed at 10 mm away and perpendicular to the plasma. The gas temperature of the plasma jets was measured by a fiber thermocouple (HQ‐FTS‐D100) with the resolution of 0.1 °C and the measuring range of 0–200 °C.

## Conflict of Interest

The authors declare no conflict of interest.

## Supporting information

Supporting Information

Supplemental Video 1
