# Supplementary material for: A Highly Cost‐Efficient Large‐Scale Uniform Laminar Plasma Jet Array Enhanced by V–I Characteristic Modulation in a Non‐Self‐Sustained Atmospheric Discharge
Source: Adv Sci (Weinh). 2020 Jan 9;7(6):1902616. doi: 10.1002/advs.201902616 (PMC7080511; doi:10.1002/advs.201902616)
Supplement: Supplementary file 1 — Supporting Information [file ADVS-7-1902616-s001.pdf]

Copyright WILEY-VCH Verlag GmbH & Co. KGaA, 69469 Weinheim, Germany, 2020.

ADVANCED SCIENCE
Open Access

Supporting Information

for *Adv. Sci.,* DOI: 10.1002/advs.201902616

A Highly Cost-Efficient Large-Scale Uniform Laminar Plasma Jet Array Enhanced by *V–I* Characteristic Modulation in a Non-Self-Sustained Atmospheric Discharge

Jing Li, Jing Wang, Bingying Lei, Tongyi Zhang, Jie Tang, Yishan Wang, Wei Zhao, and Yixiang Duan**

Supporting Information

A Highly Cost-efficient Large-scale Uniform Laminar Plasma Jet Array Enhanced by V-I Characteristic Modulation in a Non-self-sustained Atmospheric Discharge

*Jing Li, Jing Wang, Bingying Lei, Tongyi Zhang, Jie Tang, * Yishan Wang, Wei Zhao, and Yixiang Duan **

1. Proposing the Model of V-I Characteristic Modulation

1.1. Townsend's Breakdown Criterion and Paschen's Curve

When a certain voltage V is applied on a pair of parallel electrodes in a direct-current (DC) discharge cell, an approximately homogeneous electric field can be generated in the gas gap, i.e., $E = V/d$, where d is the electrode spacing. Under this condition, a photocurrent density j_0 is produced due to the cathode emission as a result of background light irradiation. In comparison with the photocurrent density at the cathode, the electron current density at the anode is enhanced by a factor $\exp(\alpha d)$, where α is the Townsend's first ionization coefficient that describes the generation of ions by electron impact. The total cathode current density possesses the same value as that of the anode electron current density and equals $j_0 \exp(\alpha d)$, which includes the electron current density j_0 and the current density of ions produced in the ionization process $j_0[\exp(\alpha d) - 1]$. With the applied voltage increased further, secondary electron emission from the cathode due to the bombarding ions comes into play and produces more ionization along the path from the cathode to the anode. The steady current density follows the form [1]

$$j = j_0 \exp(\alpha d) / \{1 - \gamma_i [\exp(\alpha d) - 1]\}, \tag{1}$$

where γ_i is the Townsend's second ionization coefficient for the cathode. As long as the denominator of Equation S1 is positive, the discharge is in a non-self-sustained state. When the denominator is negative, this equation becomes meaningless. The transition condition is that the denominator equals zero, based on which the Townsend's breakdown criterion is obtained: [2]

$$\gamma_i[\exp(\alpha d) - 1] = 1. \quad (2)$$

The Townsend's first ionization coefficient α for inert gases is related to the applied voltage V, electrode spacing d, and gas pressure p by an empirical formula [2]

$$\alpha = Cp\exp\left[-D(pd/V)^{1/2}\right], \quad (3)$$

where C and D are constants based on the gas composition.[2] Substituting Equation S3 into the breakdown criterion Equation S2, we obtain the breakdown potential

$$V_b = \frac{D^2 \cdot pd}{\left\{\ln(pd) + \ln\left[\frac{C}{\ln(1/\gamma_i + 1)}\right]\right\}^2}. \quad (4)$$

It follows from Equation S4 that the breakdown potential V_b is a function of the product of gas pressure and electrode spacing, which produces the traditional Paschen's curve, as plotted in Figure S1a. Here, the constants C and D, respectively, take the values of $29.2\,\mathrm{cm^{-1}} \cdot \mathrm{Torr^{-1}}$ and $26.6\,\mathrm{V} \cdot \mathrm{cm^{-1}} \cdot \mathrm{Torr^{-1}}$ for argon.[2] γ_i is assumed to be 0.01.[2] Figure S1a shows that in the range of relatively large pd on the right-hand branch of the curve, the breakdown potential increases almost proportionally to pd and approaches the value as high as 10190 V at $760\,\mathrm{Torr} \times 1.5\,\mathrm{cm}$.

1.2. Non-self-sustained DC Discharge

In a non-self-sustained DC discharge aforementioned, the applied voltage is considerably less than the breakdown potential and the discharge cannot be ignited in common conditions. To ignite this discharge and sustain it stably, an external ionizer is required to provide the preionization that

balances the electron losses. When the external ionizer is turned off, this discharge decays rapidly. This discharge is also named as externally sustained discharge in view of its discharge feature.[3] To demonstrate the non-self-sustained DC discharge more clearly, we consider the following system of equations describing time dependence of electrons and ions in argon:

$$\begin{cases} \frac{\partial n_e}{\partial t} = \alpha v_e n_e + \varphi - \beta n_e n_i \\ j = n_e e \mu_e E \\ v_e = \mu_e E, \ n_i \approx n_e \end{cases}, \tag{5}$$

where φ is the preionization rate of external ionizer, β is the recombination coefficient, j is the current density, e is the electron charge, E is the electric field across the gas gap, μ_e is the electron mobility, and v_e denotes the electron velocity. The electron density n_e equals the ion density n_i and these charged particles are lost mainly via recombination. For the non-self-sustained discharge mode, $\alpha v_e \ll \beta n_e$. The electron density is obtained from Equation S5 as a function of time after turning on the external ionizer:

$$n_e(t) = \left(\frac{\varphi}{\beta}\right)^{1/2} \cdot \frac{\exp\left[(\varphi\beta)^{1/2} t\right] - \exp\left[-(\varphi\beta)^{1/2} t\right]}{\exp\left[(\varphi\beta)^{1/2} t\right] + \exp\left[-(\varphi\beta)^{1/2} t\right]}. \tag{6}$$

Recently obtained experimental results show that the electron density is usually on the order of 10^{14} cm^{-3} in atmospheric-pressure argon DC discharges sustained by an external ionizer.[4–6] Under the condition that the recombination coefficient β takes a typical value of $1 \times 10^{-7} \mathrm{cm}^3 \cdot \mathrm{s}^{-1}$,[2] the preionization rate φ is estimated to be on the order of $10^{21}\,\mathrm{cm}^{-3} \cdot \mathrm{s}^{-1}$. Thus, φ can be expressed as $H \times 10^{21}\ \mathrm{cm}^{-3} \cdot \mathrm{s}^{-1}$, where $0 < H < 10$. Based on Equation S6, the temporal evolution of electron density is given in Figure S1b. It is found that the electron density $n_e(t)$ grows with time and approaches a steady value $n_s = (\varphi/\beta)^{1/2} = H^{1/2} \times 10^{14}\ \mathrm{cm}^{-3}$, when the time tends to be the characteristic time

$t_c = (\varphi\beta)^{-1/2} = \mathrm{H}^{1/2} \times 10^{-7}\ \mathrm{s}$. The discharge can be stably sustained, provided that the preionization produced by the external ionizer persists over a long time, i.e. $t_{pr} \gg t_c$. When the applied voltage between the electrodes is given, the specific energy deposited in the discharge follows the expression below

$$\omega = j \cdot E = e\mu_e \left(\frac{\varphi}{\beta}\right)^{1/2} E^2 . \tag{7}$$

Here, the electric field E is assumed to be V/d, because the cathode drop V_c under the condition of non-self-sustained DC discharge is much smaller than V and the width of layer $l_c \ll d$.[7] To sustain a discharge at a certain current density, the electric field E should obey the following equation

$$E = \left(\frac{\beta}{\varphi}\right)^{1/2} \cdot \frac{j}{e\mu_e} . \tag{8}$$

Figure S1c is a three-dimensional graph, which depicts the electric field as a function of the preionization rate φ and current density j in ranges of interest. For a given current density, the electric field decreases under conditions of strong preionization. The field, located on the red curved surface, even drops below $0.5\ \mathrm{kV/cm}$, which is much less than the breakdown field in the case of no preionization.[2] Point A represents a typical operation mode that is located on the red curved surface.

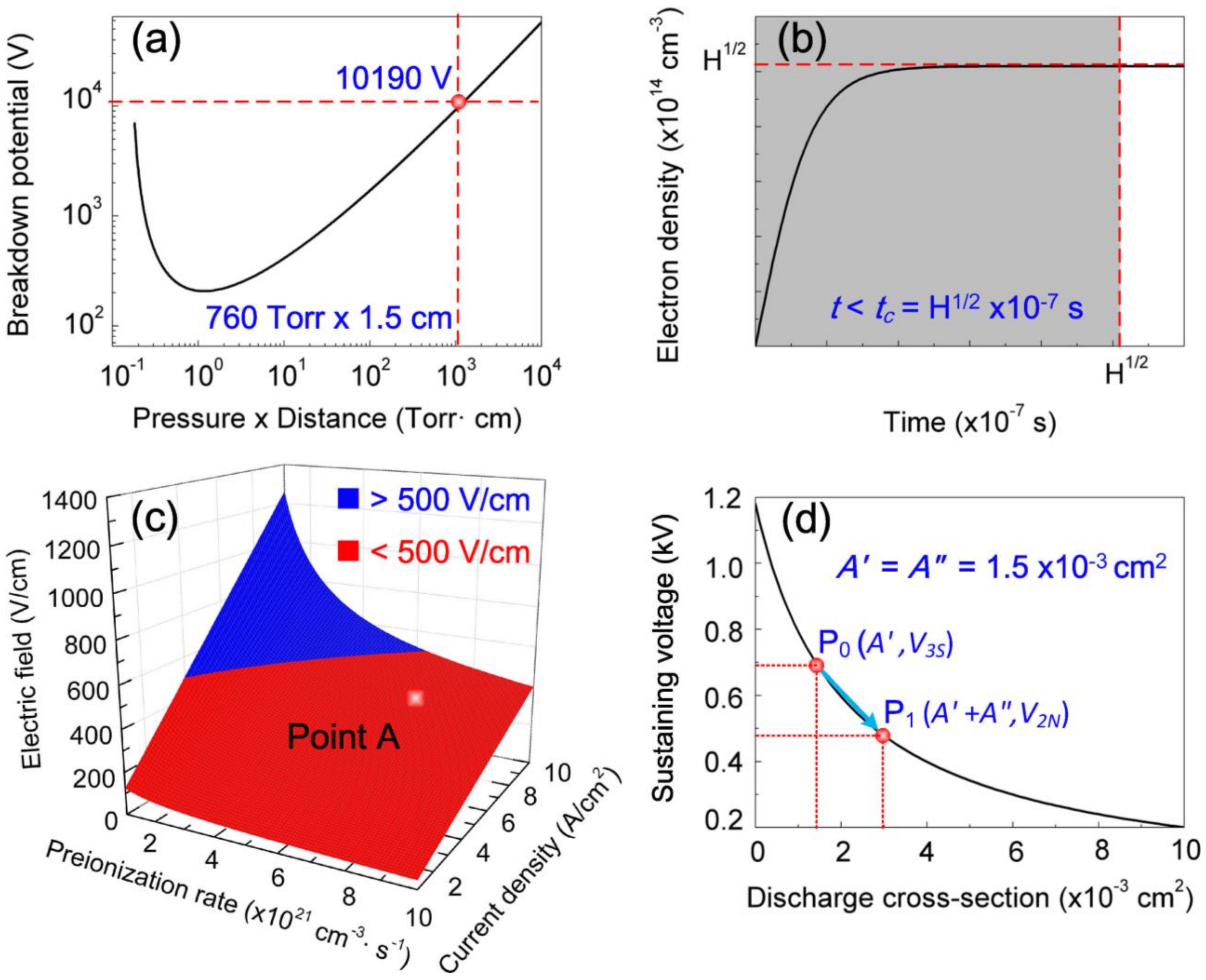

Figure S1. Electrical characteristics of DC discharge in argon. a) Breakdown potential in argon over a wide range of pd values (Paschen's curve). b) Temporal evolution of electron density in a non-self-sustained DC discharge. c) Three-dimension plot of electric field as a function of the preionization rate φ and current density j. d) Variation of the sustaining voltage with the discharge cross-section.

1.3. V-I Characteristic Modulation Model

We assume that the DC discharge cell consists of a cathode, an anode, and dielectric walls. The discharge cell is ignited and sustained by a DC power supply. Figure S2a shows the equivalent circuit for the DC discharge. The cathode is grounded and the anode is connected to the high voltage (HV) terminal of the power supply via a ballast resistor R_b that is used to limit the discharge current and

prevent the discharge from transition to an arc mode. The total capacitance C_{total} includes the capacitance of the external cable C_{cable} and the capacitance of the electrodes C_{gap}, where C_{gap} is much less than C_{cable}. The total inductance L_{total} is the sum of the external stray inductance L_s and the discharge inductance L_{dis}. For the quasisteady discharge, both the values of total capacitance and total inductance are assumed to be zero, i.e., $L_{total} = C_{total} = 0$. Thus, the discharge current I follows the equation

$$I = \left(V_a - V\right)\big/R_b = \overline{n_e}\, e\mu_e E \cdot A, \tag{9}$$

where V_a is the output voltage of the power supply, V is the sustaining voltage applied between the two electrodes, and A is the discharge cross-section area and approximately the same value as the cross sectional area of the electrodes. Here, $\overline{n_e}$ means the average value of electron density in the gas gap. It is generally accepted that with increasing the output voltage the discharge will be transformed from a Townsend discharge to a normal glow one via a subnormal region.[8] In the subnormal glow mode, the size of cathode spot is on the order of several micrometers and a thin discharge channel bridges the two electrodes.[2] Since the loss of charged particles in the lateral direction is harmful for multiplication due to free diffusion of electrons to the side walls, the average electron density is relatively low. To increase the average electron density for high plasma chemical activity in practical applications, the discharge is usually allowed to work in a normal or abnormal mode by further raising the output voltage. But this manipulation brings about another harmful factor, i.e., generation of considerable Joule heat both in the discharge cell and in the ballast resistor because of the increase in the discharge current. The most desirable way is realization of a normal or abnormal glow discharge at a smaller discharge current.

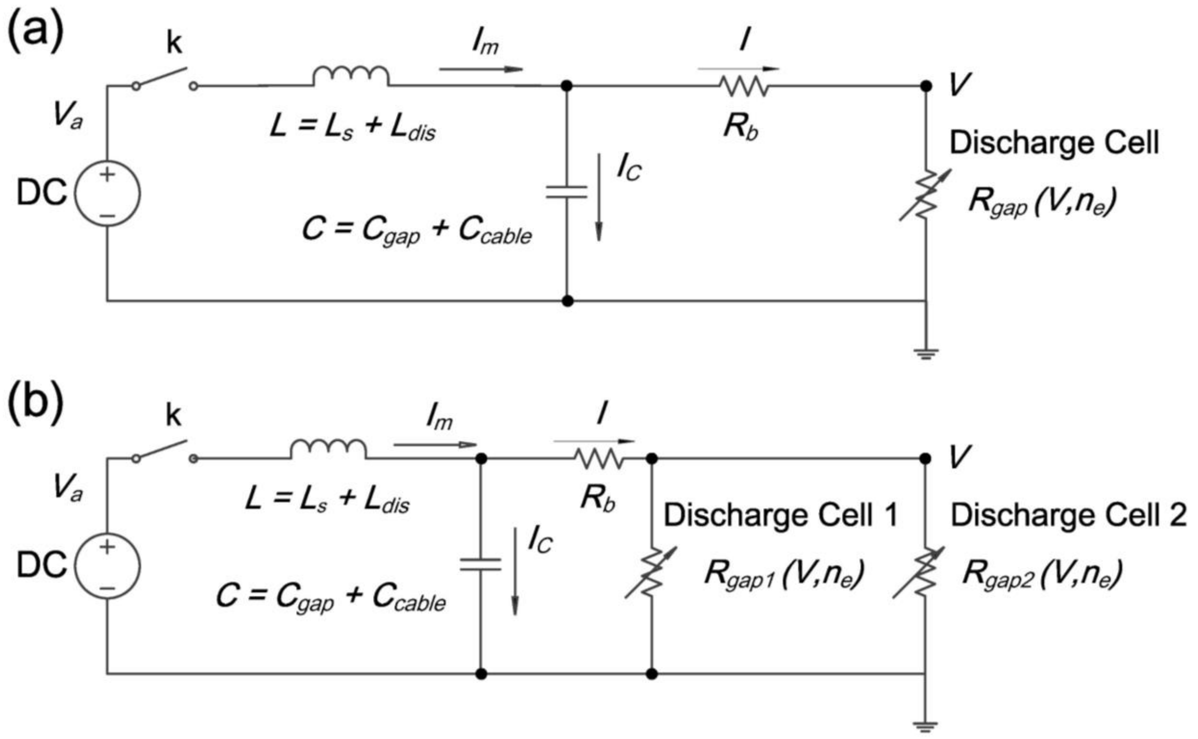

Figure S2. Schematic diagram of equivalent circuit models. a) Model for a single DC discharge cell. b) Model for two DC discharge cells arranged in parallel.

To explore this method, we substitute the equation $E = V/d$ into Equation S9 and obtain

$$V = \frac{V_a d}{d + A \cdot \overline{n_e} e \mu_e \cdot R_b}. \tag{10}$$

It follows from Equation S6 that in the non-self-sustained DC discharge, the electron density, as well as its average value $\overline{n_e}$, is largely determined by the preionization rate φ and changed little in the subnormal transition region.[7] Thus, when the output voltage V_a and ballast resistor R_b are given, the sustaining voltage V will decrease with increasing the discharge cross-section area, with the relationship shown in Figure S1d. Here, in order to conveniently compare with the experimental results, we assign 1180 V to V_a, 50 kΩ to R_b, 1.85×10^{14} cm^3 to $\overline{n_e}$, and 1.5 cm to d by referring to the experimental electrical parameters at the operating point 3S demonstrated in Figure 5c in the main text.

It should be noted that the reduction of sustaining voltage with an approximate constant electron density means that loss of charged particles in the lateral direction slows down and the discharge is likely to transform from the subnormal mode to a normal one. The cross-section increase can be fulfilled by enlarging the cross-section of the discharge cell itself. Additionally, arranging several discharge cells in parallel is another effective way to increase the discharge cross-section. Two DC discharge cells arranged in parallel is the most simple case. Figure S2b shows its equivalent circuit. The total discharge current and sustaining voltage are expressed as follows

$$\begin{cases} I = \left(V_a - V\right)/R_b = \left(A' + A''\right) \cdot \overline{n_e} e\mu_e E \\ V = \dfrac{V_a d}{d + \left(A' + A''\right) \cdot \overline{n_e} e\mu_e \cdot R_b} \end{cases}, \tag{11}$$

where A' and A'', respectively, represent the discharge cross-sections of the two discharge cells and $A' = A'' = 1.5 \times 10^{-3}\ \mathrm{cm}^2$. From Figure S1d, it follows that when the discharge cross-section increases from A' to $A' + A''$ through the parallel connection, the sustaining voltage in the two discharge cells will be abruptly reduced from $V_{3S} = 680$ V to $V_{2N} = 478$ V. The operating point shifts from P_0 to P_1. Here, we assume that the discharge at a higher sustaining voltage V_{3S} is a subnormal one, which occurs at a smaller discharge current I_{3S}. It is generally recognized that the discharge is likely to extinguish due to the abrupt reduction of sustaining voltage in a self-sustained discharge. But in the non-self-sustained DC discharge, discharge sustainment is ensured by seed charges from the external ionizer.

It should be noted that the reduction of sustaining voltage in the discharge with an approximate constant electron density means that loss of charged particles in the lateral direction slows down and the discharge is likely to transform from the subnormal mode to a normal one. Referring to the equation $I = \left(V_a - V\right)/R_b$, we find that the total discharge current I increases in the parallel circuit. But, the discharge current flowing through each discharge cell does not increase but decreases to

some extent, which is ascribed to the decreasing electric field between the electrodes as a result of the decreasing sustaining voltage. This current decrease can be demonstrated by Equations S8 and S9. Thus, we can obtain the following relationship

$$\begin{cases} I_{3S} < I_{2N} < 2I_{3S} \\ I_{3S} = A' \cdot \overline{n_{e3S}} e\mu_e E_{3S} \\ I_{2N} = \left(A' + A''\right) \cdot \overline{n_{e2N}} e\mu_e E_{2N} \end{cases}, \tag{12}$$

where I_{2N} is the sum of discharge current of the two discharge cells in the parallel circuit. For each of the discharge cells, the discharge current equals $I_{2N}/2$. To sustain a discharge with the initial value I_{3S}, it is necessary to increase the discharge current. This current increase is realized by raising the output voltage V_a, which can be explained as follows.

Although the discharge with the sustaining voltage V_{2N} and current $I_{2N}/2$ for each discharge cell is operated in the normal or quasi-normal glow mode, the luminous current spot on the cathode surface is still limited on the order of several micrometers.[2] With increasing the output voltage V_a, the electron density and current density remain constant in the discharge channel, but the luminous current spot on the cathode surface expands outwards and the discharge channel occupies more portions of the gas gap.[2] After the discharge covers the entire cathode surface, the discharge transforms to the abnormal mode and any further increase of the output voltage increases the current density in compared with the normal value.[2] Either the expansion of discharge channel in the normal mode or the increase of current density in the abnormal regime inevitably increases the discharge current in the gas gap.

It is concluded that the V-I characteristic modulation model proposed above includes two key steps, i.e., 1) arranging non-self-sustained DC discharge cells in parallel with the output voltage unvaried, and 2) raising the output voltage until the discharge current increases to the initial value.

This model succeeds in realizing a normal or abnormal glow discharge with a higher average electron density at a smaller discharge current, which creates a new road map for the enhanced gas discharge basic theory.

2. Principle of the DBD Preionization

Figure S3a shows the vertical view of the laminar plasma jet array (LPJA) device. The left and right edges of discharge region are marked by using green arrows, where two ground electrodes of DC discharge are located. The dielectric-barrier discharge (DBD) occurs between the sidewalls in the ceramic discharge chamber under the condition that the DC voltage is zero. Figure S3b shows a typical image of DBD plasma with the argon flow fixed at 13 l/min. The plasma presents two intensive luminous layers close to the sidewalls and distributes uniformly along the x axis, except that a stronger optical emission appears at the four ground electrodes of DC discharge (x = -45 mm, -15 mm, 15 mm, and 45 mm). This means that the ground electrodes set downstream are able to enhance the DBD to some extent in the local regions. The physical appearance of DBD occurring along the x axis from 15 to 45 mm is enlarged in Figure S3c. It is likely that a diffuse plasma is achieved in the DBD. But the waveforms of applied voltage $u(t)$ and discharge current $i(t)$ (the total current subtracting the displacement current), as shown in Figure S3d, indicate that the DBD operates in a filamentary mode, because the width of current pulses is about 360 ns and much less than that (several to tens of μs) observed in the glow-like discharge.[9,10] From Figure S3d, it is also found that only one current pulse occurs each half cycle with its amplitude ranging from 10 to 30 mA, when the peak voltage u_{peak} is set at 2.5 kV. The electric power p_{DBD} dissipated in the DBD is determined by Manley's theory:[11]

$$p_{DBD} = 4fC_d u_{dis}(u_{peak} - \frac{C_d + C_g}{C_d} u_{dis}) . \qquad (13)$$

Here, u_{dis} is the average discharge voltage across the gas gap, while C_d and C_g are the capacitances of dielectrics and gas gap, respectively. The waveforms of applied voltage and discharge current indicate that the DBD stops before the peak voltage in the former positive half cycle and does not occur until the applied voltage approaches about -1.62 kV in the latter negative half cycle. The threshold voltage u_{th} is determined to be -1.62 kV and related to the average discharge voltage by

$$u_{th} = u_{peak} - 2(C_d + C_g)C_d^{-1}u_{dis}. \tag{14}$$

With $C_d = 27.4\,\mathrm{pF}$ and $C_g = 8.9\,\mathrm{pF}$, the average discharge voltage and the electric power are respectively estimated to be 1.45 kV and 610 mW. This low electric power is used to improve the downstream DC discharge.

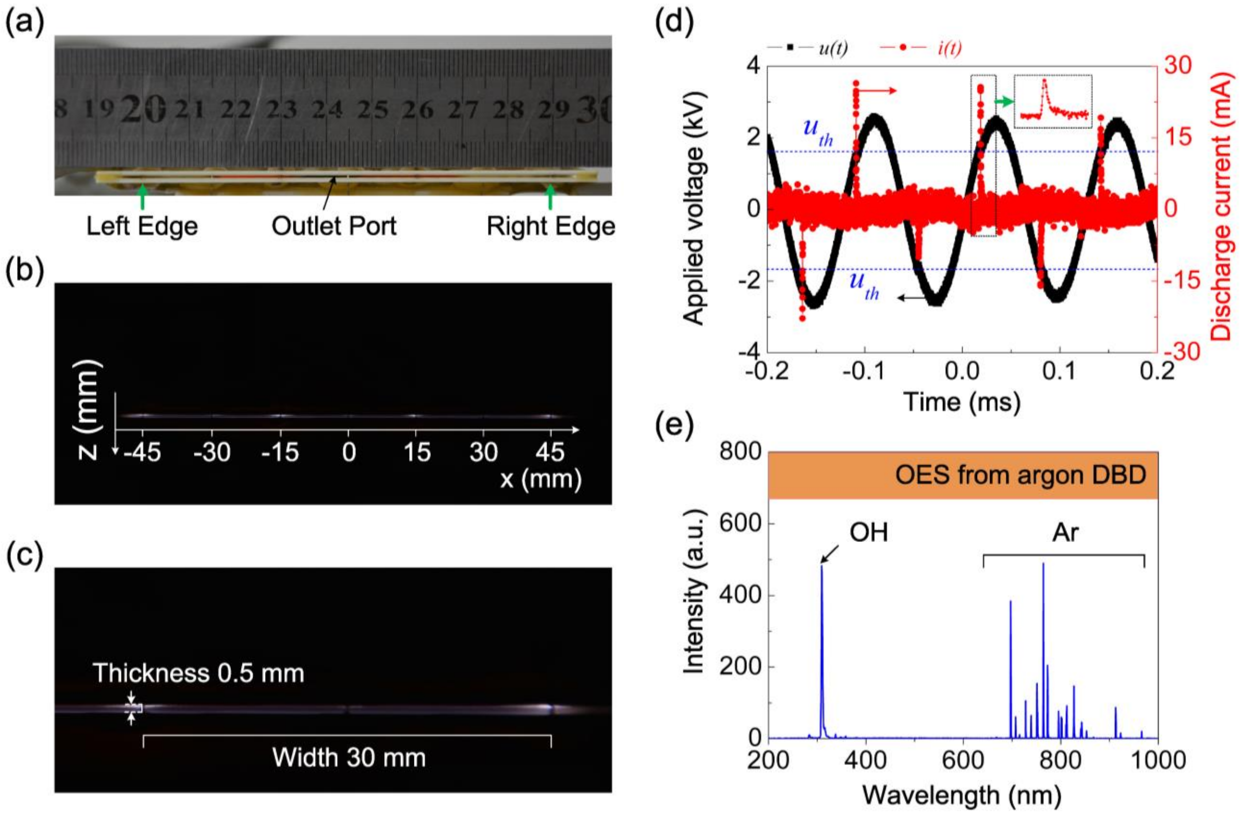

Figure S3. Optical and electrical characteristics of the argon DBD. a) The physical map of the LPJA from the vertical view. b) Image of the argon DBD between the sidewalls in the ceramic discharge

chamber. c) Enlarged image of the argon DBD that occurs along the x axis from 15 to 45 mm. d) The waveforms of the applied voltage and discharge current for the argon DBD. e) The OES from the argon DBD.

As for the DBD, electrons and argon ions are generated in the preionization space. The drift velocity for electrons and ions can be approximately determined by

$$v_{e,i} = \mu_{e,i} E_{DBD}, \tag{15}$$

where the electron mobility μ_e and ion mobility μ_i are assumed to be $4.3 \times 10^2\ \mathrm{cm^2/(V \cdot s)}$ and $1.9\ \mathrm{cm^2/(V \cdot s)}$, respectively.[12] E_{DBD} means the average electric field, i.e., the average discharge voltage u_{dis} divided by the gas gap. With the gas gap of 0.5 mm and average discharge voltage of 1.45 kV, E_{DBD} is estimated to be 29 kV/cm. Substituting these values into Equation S15, $v_e = 1.2 \times 10^7\ \mathrm{cm/s}$ and $v_i = 5.5 \times 10^4\ \mathrm{cm/s}$. Both the electron drift velocity and the ion drift velocity are much higher than the gas flowrate $v_g = 4.3 \times 10^1\ \mathrm{cm/s}$. This means that few charged particles generated in the DBD can be brought downstream to the DC discharge space by the feeding gas flow and that the contribution of these charged particles to the downstream DC discharge is negligible.

Figure S3e shows the optical emission spectra (OES) from the DBD. Argon emission lines from the 4p→4s transitions are dominantly presented in the visible and infrared region between 690 and 950 nm due to the argon feeding gas. In addition, strong OH emission line at 309 nm is also observed because of the gas impurity vapor. The OES indicates that sufficient excited argon atoms exist in the discharge. It has been reported that the lifetime of argon metastables can reach up to several seconds.[2] Since tens of milliseconds is required for the gas to flow from the DBD region to the DC discharge space, many argon metastables will arrive at the downstream DC discharge space and ionizations due to collisions between argon metastables are able to efficiently create new charged

particles there by the Penning effect [13]

$$Ar(4s) + Ar(4s) \rightarrow Ar + Ar^{+} + e. \qquad 6.4 \times 10^{-10}\ cm^3s^{-1} \tag{16}$$

These charged particles, served as seed charges, accelerate the formation of electron avalanche and enhance the ionization efficiency of the DC discharge. Additionally, the secondary electron emission from a cold cathode is caused by positive ions, excited atoms, electrons, and photons. Among them, the excited atoms coming from the DBD are very efficient in the DC discharge.[2] Due to the enhancement of ionization efficiency, the DC discharge can be initiated in a large gas gap (15 mm) far below the Townsend breakdown potential. Thus, we conclude that the Penning ionization of argon metastables from the DBD plays a crucial role in improvement of the downstream DC discharge.

3. Enhancement of the Plasma Chemical Activity

Since the LPJA is composed of three independent discharge units, as illustrated in Figure 2c in the main text, each of the plasma jets in the discharge unit shows a similar or same optical emission characteristic to that in the array. Thus, Case 2 shown in Figure 6b in the main text was selected as alternative to make a comparative OES examination in contrast to Case 5 shown in Figure 6i in the main text by using a moderate-resolution spectrometer. It should be noted that the two-arrayed plasma jet shown in Figure 6i is composed of two independent discharge cells and its V-I characteristic (Not shown here) is similar to that of Case 3 shown in Figure 6g in the main text. The coordinate origin is set at the transverse center of the two-arrayed plasma jet and just above the outlet of the discharge chamber in the two cases. Figure S4a shows the OES detected in Case 2 and Case 5 at a specific point with the relative position x = 7.5 mm and y = 1 mm in accordance with the axes labeled in Figure 2a in the main text. For both the cases, various argon emission lines are dominantly

presented due to the argon feeding gas in the visible and infrared region between 690 and 950 nm. Additionally, the OES from the $OH(A\,^2\Sigma^+ \rightarrow X\,^2\Pi)$ transition and the nitrogen second positive system $N_2(C\,^3\Pi_u \rightarrow B\,^3\Pi_g)$ transition are both observed in the ultraviolet and visible region between 200 and 450 nm.

As we know, the energy carried by the excited argon atoms (11.5–13.5 eV) is slightly higher than the energy level of $N_2(C\,^3\Pi_\mu)$ (~ 11.1 eV).[14,15] Energy is easily transferred from the excited argon atoms to the ground state nitrogen molecules in the ambient air to generate abundant excited state molecules $N_2(C\,^3\Pi_\mu)$. This process follows the reaction 1: $N_2(X) + Ar(4p) / Ar(4s) \rightarrow N_2(C) + Ar$ with the rate of 10^{-11}-10^{-10} cm^3s^{-1}.[16] In addition, the energy required to dissociate H_2O from the impurity in the feeding gas and the ambient air into OH and H, and subsequently excite the ground state OH to the excited state $OH(A\,^2\Sigma^+)$ is, respectively, 5.1 and 4.2 eV.[14] Sufficient excited state molecules $OH(A\,^2\Sigma^+)$ are generated by energy transfer from the excited argon atoms. The related reaction 2 is expressed as $H_2O + Ar(4p) / Ar(4s) \rightarrow OH(A) + H + Ar$ with the rate of $\sim 10^{-10}$ cm^3s^{-1}.[16] Thus, the OES originated from the excited OH and N_2 are clearly presented in the plasma jets, even if the electron-impact excitation is not fully ensured. It should be noted that the OH radical has a strong oxidative effect on the cell outer structure and is identified as a major contributor in the plasma inactivation. Comparing the spectra in the two cases shows that the spectral intensity of the plasma generated in the normal/abnormal glow discharge is approximately increased by two times compared to that in the subnormal glow discharge. This means that the concentration of reactive species in the plasma, as well as the plasma chemical activity, can also be enhanced by modulating the V-I characteristics of DC discharge.

The spatial distributions of OES from OH, N_2, and Ar in the plasma produced in the two cases

were also examined at y = 1 along the x axis with the results shown in Figure S4b–d. For the two two-arrayed plasma jets, all the reactive species present a near-symmetrical distribution of emission profile with respect to the central axis with the intensity greater near the electrodes than in the positive column. The greater intensity near the electrodes is attributed to the negative glow and anode glow. This symmetrical feature remains unchanged for each plasma jet in the normal/abnormal glow discharge (Case 2), but is broken for each one in the subnormal glow discharge (Case 5), where the intensity is greater at the cathode than at the anode. An approximately flat area is presented between ±4 and ±11 mm on the x-axis, which suggests that the reactive species are distributed uniformly in the middle part of the plasma jet. Comparison of the spatial distributions of OES in the two cases indicates that these reactive species in the gas gap, especially in the positive column, have a higher concentration in Case 2 than in Case 5.

Since the central part of each plasma jet in Case 5 just extends out of the discharge chamber outlet, the spatial distributions of OES from reactive species have not been examined. Figure S4e, f show the spectral intensity varies along the y direction with x = ± 7.5 mm only for Case 2. It is found that a similar or same spatial emission profile is presented for the same reactive species at x = ± 7.5 mm. The spectral intensity of Ar first declines slowly near the outlet ($y < 1\,\mathrm{mm}$), then drops quickly with increasing the distance from the outlet ($1\,\mathrm{mm} \le y < 3\,\mathrm{mm}$), and finally tends to be zero when the length increases further than 3 mm. But for OH and N_2, the emission intensities first rise, approach their maximum at y = 1 mm, then fall, and fade away at the length of 4 mm or greater. The spectral intensity of Ar prevails against others all along the length, with the OH spectral intensity remaining weakest throughout the region. Characterizing the spatial distributions of OES suggests that most of the reactive species assemble in the vicinity of the nozzle ($y \le 2\,\mathrm{mm}$). The fast degradation of Ar

spectral intensity along the length is mainly due to the high quenching rates by vapor, nitrogen molecules, and oxygen molecules from the ambient air (Reaction 1, Reaction 2, and Reaction 3: O_2 + Ar(4p) / Ar(4s)→products with the rate of 10^{-11}-10^{-10} cm^3s^{-1}).[15] The distinct spatial emission profiles of OH, N_2, and Ar are attributed to the different generation and quenching mechanisms of their corresponding excited states, i.e., $OH(A\ ^2\Sigma^+)$, $N_2(C\ ^3\Pi_\mu)$, and Ar(4p)/Ar(4s) in the plasma jets.

The light emitted from the plasma is originated from the energy transition of species from the excited state to the lower or ground one. For Case 1, after ejecting out of the outlet, the argon plasma jets interact with the ambient air. The interaction processes are closely associated with the dynamic behavior of flowing feeding gas, mixing characteristics of feeding gas and ambient air, and the different generation and quenching mechanisms of excited species in the plasma.[15,17] Due to the flat duck-mouth structure of the discharge chamber and the central single loop gas supply mode, the argon flow rate is not strictly uniformly distributed along the transverse direction, but with greater values in the center than at the edges, which is indirectly reflected by the spatial distribution of light intensity along the transverse direction at y = 0 mm shown in Figure 4b in the main text. Thus, more excited argon atoms are gathered in the central region of the LPJA, where the interaction between the argon plasma with the ambient air is more intense through Reactions 1, 2, and 3. Since the spectral intensity of Ar remains dominant all along the length, as shown in Figure S4e, f, more intense reactions occurring in the center give a chance to degrade the light intensity in a greater degree there. This is the possible reason for the transverse profile evolution of light intensity with the length depicted in Figure 4b.

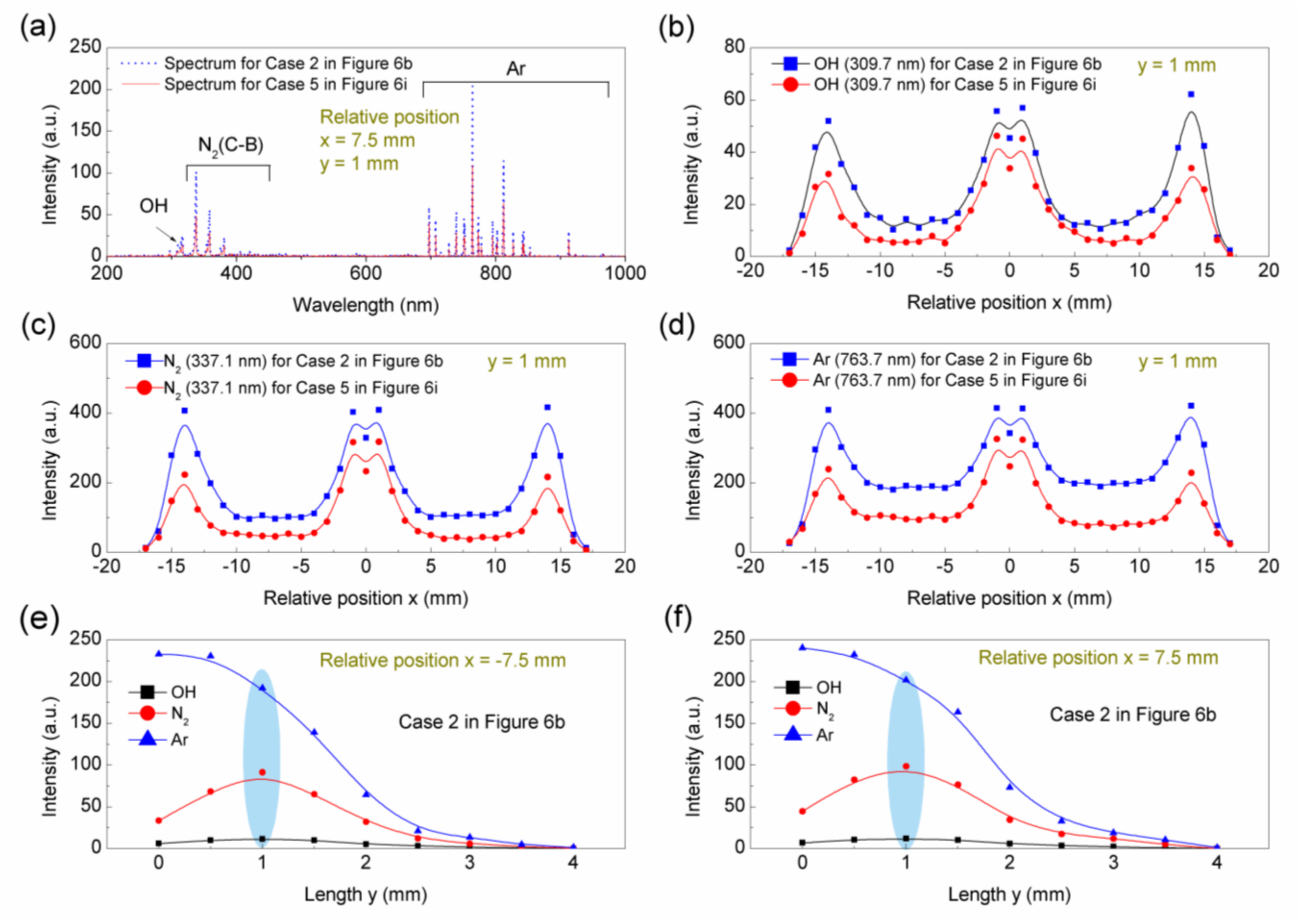

Figure S4. Spectral enhancement of the LPJA. a) Spectral intensity comparison between Cases 2 and 5 at a specific point with the relative position x = 7.5 mm and y = 1 mm. b) Transverse distributions of spectral intensity of OH for Cases 2 and 5 at y = 1 mm. c) Transverse distributions of spectral intensity of N_2 for Cases 2 and 5 at y = 1 mm. d) Transverse distributions of spectral intensity of Ar for Cases 2 and 5 at y = 1 mm. e) Spatial distributions of spectral intensity of OH, N_2, and Ar for Case 2 along the length with the relative position x = -7.5 mm. f) Spatial distributions of spectral intensity of OH, N_2, and Ar for Case 2 along the length with the relative position x = 7.5 mm.

4. Nonequilibrium Characteristics of the LPJA

Nonequilibrium characteristics of the plasma were verified for Case 1 shown in Figure 4a in the main text by comparing the rotational temperature (T_{rot}) and vibrational temperature (T_{vib}) of the plasma. The coordinate origin is set at the transverse center of the LPJA and just above the outlet of the

discharge chamber. Based on the emission spectrum recorded by a high-resolution spectrometer at the point with x = 7.5 mm and y = 1 mm, the rotational temperature and vibrational temperature of the LPJA were obtained by analyzing the OES of OH and N_2 second positive system, respectively.[18–21] Figure S5a shows the best-fitting synthetic spectrum to the experimental spectrum for the $OH(A\,^2\Sigma^+ \rightarrow X\,^2\Pi, \Delta v = 0)$ band transition from 306 to 312 nm. The rotational temperature T_{rot} is determined to be about 640 K. As for the vibrational temperature, this spectrum fitting method was applied to the spectral profile of the nitrogen second positive system $N_2(C\,^3\Pi_\mu \rightarrow B\,^3\Pi_g, \Delta v = \text{-}2)$ from 370 to 382 nm, with the result shown in Figure S5b. This temperature T_{vib} is estimated to be about 2630 K and much higher than the rotational one. Comparison of the rotational and vibrational temperatures indicates that the LPJA is under nonequilibrium condition, which contributes much to the enhancement of plasma chemistry.

As mentioned above, the energy transfer from excited argon atoms plays an important role in production of the excited $OH(A\,^2\Sigma^+)$. It is unsuitable to determine the gas temperature through the estimation of the rotational temperature of OH due to its overpopulation at high rotational states.[15] Thus, a fiber thermocouple was used to measure the gas temperature of the LPJA at y = 1 mm along the x direction. Figure S5c shows the transverse spatial distribution of the gas temperature. It is found that the gas temperature is periodically distributed in space due to the periodic electrode structure. Different gas temperatures are observed near the electrodes, with the value approaching 120 ℃ at the cathode but only about 88 ℃ at the anode. The lowest gas temperature is approximately 70 °C in the central part of each plasma jet. Additionally, the gas temperature was also examined along the length at x = 7.5 mm, with the result shown in Figure S5d. It is found that the gas temperature first declines slowly and remains about 70 °C near the nozzle ($z \leq 2$mm), where

relatively more reactive species are accumulated. Then, the gas temperature is decreased with a greater rate with increasing the distance from the outlet. Finally, it falls to 63 °C at the length of 4 mm, where the excited OH, N_2, and Ar nearly disappear. Spatial examination of the LPJA gas temperature shows that this temperature is far less than the rotational temperature, but close to the room temperature, which is beneficial to treating samples that are susceptible to high temperatures.

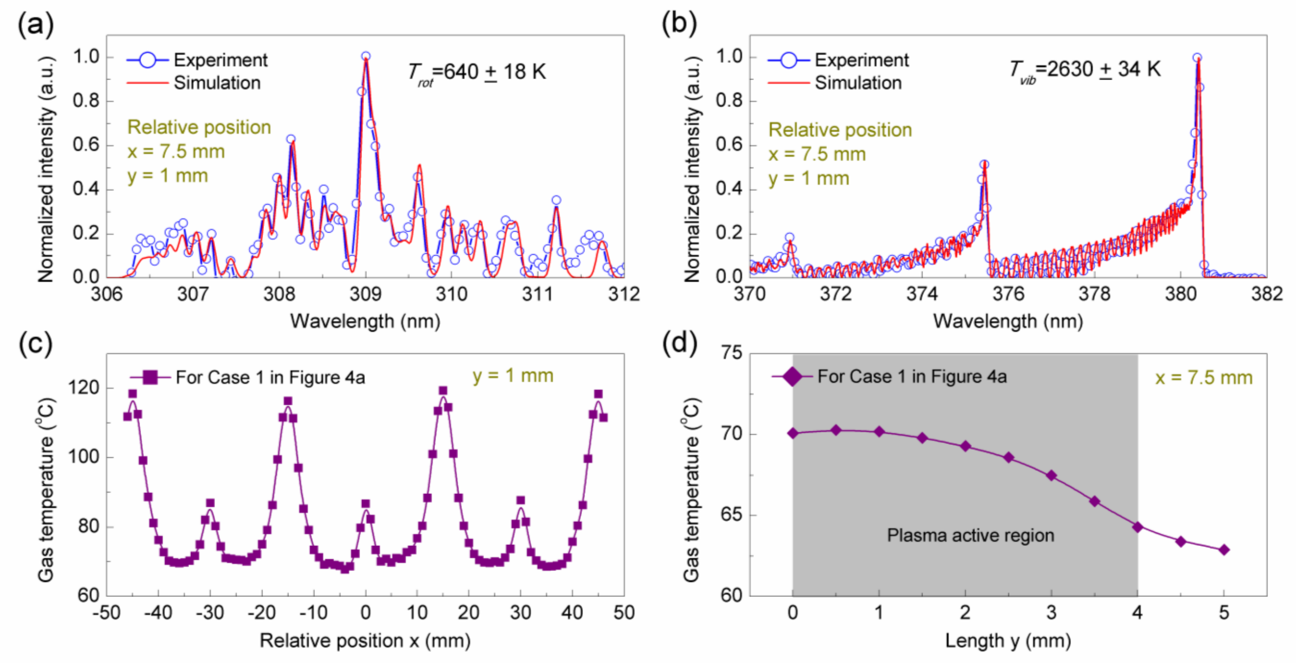

Figure S5. Nonequilibrium characteristics of the LPJA shown in Figure 4a in the main text. a) Determination of the rotational temperature T_{rot} by comparing experimental and simulated spectra from $OH(A\ ^2\Sigma^+ \rightarrow X\ ^2\Pi)$ transition. b) Determination of the vibrational temperature T_{vib} by comparing experimental and simulated spectra from $N_2(C\ ^3\Pi_u \rightarrow B\ ^3\Pi_g)$ transition. c) The transverse distribution of gas temperature at y = 1 mm. d) The spatial distribution of gas temperature along the length at x = 7.5 mm.

REFERENCES

[1] D. B. Go, D. A. Pohlman, *J. Appl. Phys*. **2010**, *107*, 103303.

[2] Y. P. Raizer, *Gas Discharge Physics*, Springer, Berlin, Germany, **1991**.

[3] E. P. Velikhov, S. A. Golubev, Yu. K. Zemtsov, A. F. Pal, I. G. Persiantsev, V. D. Pismennyi, A. T. Rakhimov, *Sov. Phys.-JETP* **1974**, *38*, 267.

[4] J. Tang, S. Li, W. Zhao, Y. Wang, Y. Duan, *Appl. Phys. Lett*. **2012**, *100*, 253505.

[5] X. Li, J. Tang, X. Zhan, X. Yuan, Z. Zhao, Y. Yan, Y. Duan, *Appl. Phys. Lett*. **2013**, *103*, 033519.

[6] W. Jiang, J. Tang, Y. Wang, W. Zhao, Y. Duan, *Appl. Phys. Lett*. **2014**, *104*, 013505.

[7] G. A. Mesyats, Y. D. Korolev, *Sov. Phys. Usp*. **1986**, *29*, 57.

[8] R. R. Arslanbekov, V. I. Kolobov, *J. Phys. D: Appl. Phys*. **2003**, *36*, 2986.

[9] B. Qi, C. S. Ren, D. Z. Wang, S. Z. Li, K. Wang, Y. T. Zhang, *Appl. Phys. Lett*. **2006**, *89*, 131503.

[10] F. Massines, A. Rabehi, P. Decomps, R. B. Gadri, P. Segur, C. Ayoux, *J. Appl. Phys*. **1998**, *83*, 2950.

[11] U. Kogelschatz, *Plasma Chem, Plasma Proc*. **2003**, *23*, 1.

[12] A. D. Richards, B. E. Thompson, H. H. Sawin, *Appl. Phys. Lett*. **1987**, *50*, 492.

[13] K. Kutasi, V. Guerra, P. A. Sa, *Plasma Sources Sci. Technol*. **2011**, *20*, 035006.

[14] Y. Xian, X. Lu, Z. Tang, Q. Xiong, W. Gong, D. Liu, Z. Jiang, Y. Pan, *J. Appl. Phys*. **2010**, *107*, 063308.

[15] Q. Xiong, A. Nikiforov, X. Lu, C. Leys, *J. Phys. D: Appl. Phys*. **2010**, *43*, 415201.

[16] J. E. Velazco, J. H. Kolts, D. W. Setser, *J. Chem. Phys*. **1978**, *69*, 4357.

[17] X. Y. Liu, X. K. Pei, X. P. Lu, D. W. Liu, *Plasma Sources Sci. Technol*. **2014**, *23*, 035007.

[18] S. Moon, W. Choe, *Appl. Phys. Lett*. **2004**, *84*, 188.

[19] X. Li, P. Zhang, P. Jia, J. Chu, J. Chen, *Sci. Rep*. **2017**, *7*, 2672.

[20] J. Li, Y. Xu, T. Zhang, J. Tang, Y. Wang, W. Zhao, Y. Duan, *J. Appl. Phys*. **2017**, *122*, 013301.

[21] C. O. Laux, T. G. Spence, C. H. Kruger, R. N. Zare, *Plasma Sources Sci. Technol*. **2003**, *12*, 125.
